# Supplementary material for: Morpholino-mediated splice switching of NELFE suppresses hepatocellular carcinoma cell phenotypes in vitro and in vivo
Source: Mol Ther Nucleic Acids. 2026 Oct 1;37(4):103124. doi: 10.1016/j.omtn.2026.103124 (PMC13694868; doi:10.1016/j.omtn.2026.103124)
Supplement: Supplementary file 1 [file mmc1.pdf]

OMTN, Volume *37*

Supplemental information

Morpholino-mediated splice switching of NELFE suppresses hepatocellular carcinoma cell phenotypes *in vitro* and *in vivo*

Laura M. Reynolds, Ryan Lamm, Kai Zhang, Anna E. Barry-Wolbers, Alvaro Lucci, Brittany N. Ruiz, Corinne E. Wessner, Trish Nguyen, Kayla M. Hammond, Christoph Eckert, Ji-Bin Liu, Pongsakorn Choochuen, Kai Ying Hong, Wei Jiang, Marshonna Forgues, Gaia M. Palazzo, Daniel Lin, Adam S. Bodzin, Ashesh P. Shah, Xin Wei Wang, Sean P. Martin, Nicolas L. Fawzi, Matthias M. Gaida, John R. Eisenbrey, and Hien Dang

Supplemental Materials

Characteristic	WT (n=73)	SNP (n=8)
Age		
Mean (SD)	52.6 (13.3)	56.0 (8.6)
BMI		
Mean (SD)	23.0 (3.4)	24.9 (2.2)
Pre-op ALT (IU/L)		
Mean (SD)	77.3 (136.5)	41.0 (12.2)
Pre-op AFP (ng/mL)		
Mean (SD)	12,268.3 (22,606.1)	160.0 (423.1)
Main Tumor Size (cm)		
Mean (SD)	7.11 (3.44)	4.23 (1.80)
Gender		
Male	65 (89%)	6 (75%)
Female	8 (11%)	2 (25%)
HBV		
Yes	69 (94.5%)	7 (87.5%)
No	4 (5.5%)	1 (12.5%)
HCV		
Yes	2 (2.7%)	1 (12.5%)
No	71 (97.3%)	7 (87.5%)
Cirrhosis		
Yes	55 (75.3%)	7 (87.5%)
No	18 (24.7%)	1 (12.5%)
Surgical Procedure		
Transplant	5 (6.8%)	0 (0%)
Resection	68 (93.2%)	8 (100%)
Previous Treatment		
Yes	8 (11%)	0 (0%)
No	65 (89%)	8 (100%)
Satellite Nodule		
Yes	14 (19.2%)	1 (12.5%)
No	59 (80.8%)	7 (87.5%)
Multinodular		
Yes	24 (32.9%)	1 (12.5%)
No	49 (67.1%)	7 (87.5%)
Invasion of Lymph Nodes		
Yes	0 (0%)	0 (0%)
No	73 (100%)	8 (100%)
TNM Stage		
I	40 (54.8%)	8 (100%)
II	10 (13.7%)	0 (0%)
III	23 (31.5%)	0 (0%)
IV	0 (0%)	0 (0%)

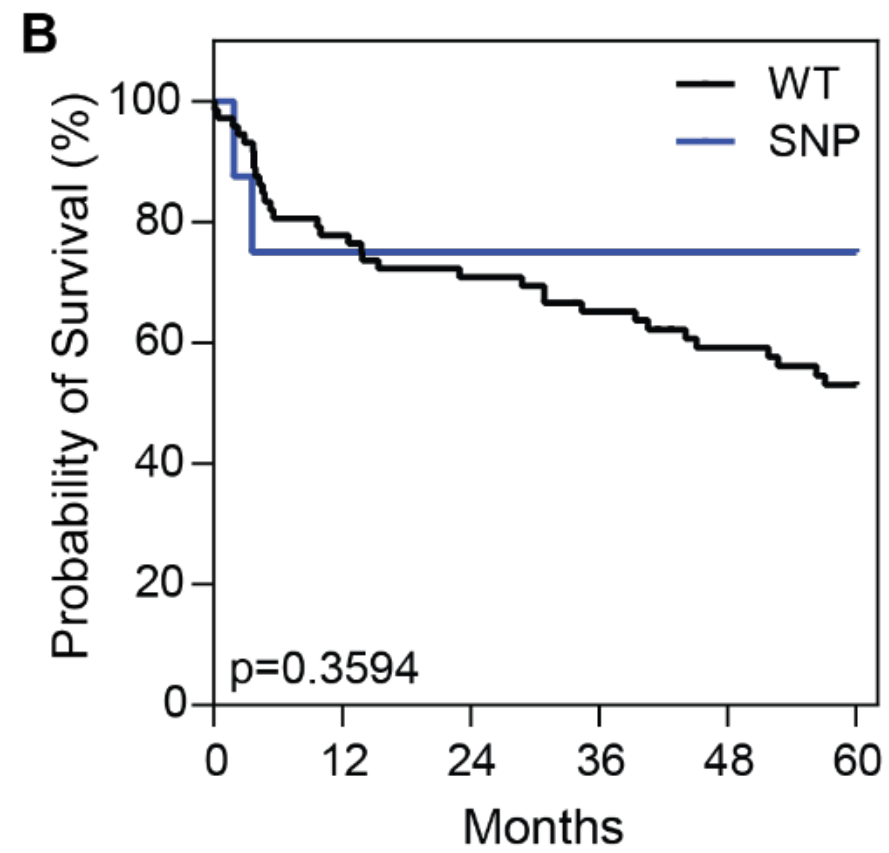

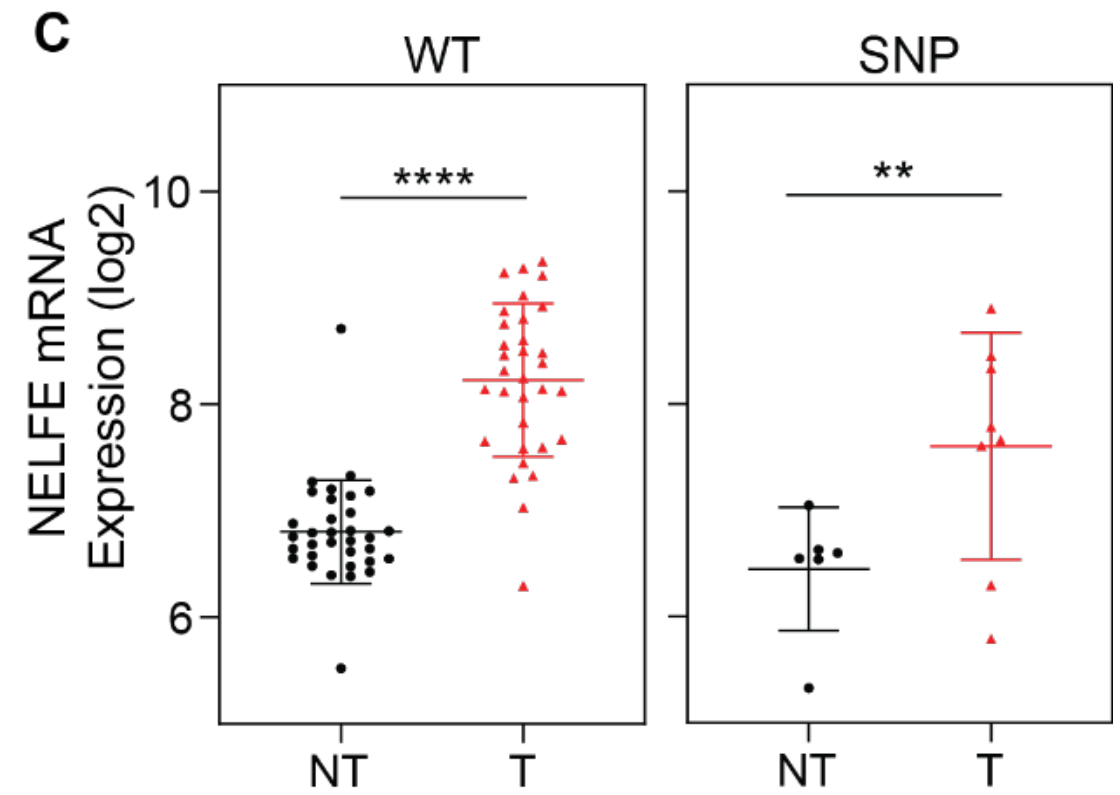

Figure S1. Rs79208225 SNP in HCC patients is associated with decreased *NELFE* mRNA expression. (A) Demographic and clinical variables of WT and rs79208225 SNP patient cohorts. **(B)** Kaplan-Meier 5-year overall survival curve for WT and rs79208225 SNP patients (WT n=73, SNP n=8). Statistical analysis was conducted by log-rank test. **(C)** TaqMan RT-qPCR to detect *NELFE* mRNA expression between tumor (T) and non-tumor (NT) tissue in WT (T n=32, NT n=33) and SNP (T n=8, NT n=6) patients. Data presented as mean±SD. Statistical analysis was conducted with a one-way ANOVA with Holm-Šídák's multiple comparisons test. **p< 0.01, ****p<0.0001

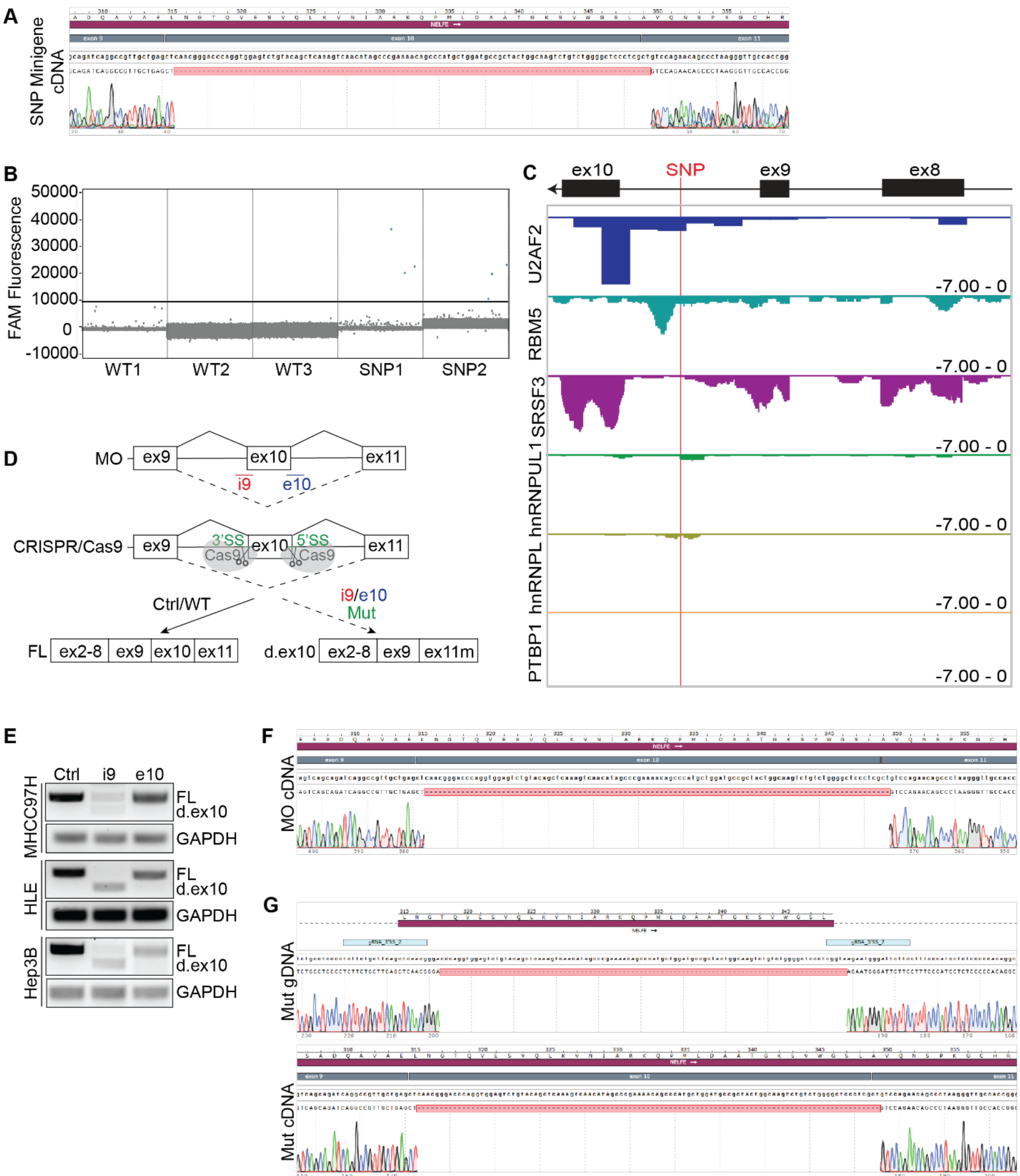

Figure S2. Rs79208225 SNP promotes alternative splicing of *NELFE* exon 10 producing an uncharacterized *NELFE* mRNA isoform. (A) Sanger sequencing of the bottom band from the SNP minigene RT-PCR in Fig. 2A showing exon 10 skipping. **(B)** Digital PCR data of d.ex10 isoform detection in WT and rs79208225 SNP patient tumor tissue. Each dot is a well. Threshold (black line) is 9500. **(C)** Crosslinking immunoprecipitation data from the ENCODE database of splicing related proteins visualized using integrative genomics viewer to identify proximity of their

binding regions to the rs79208225 locus (red line). **(D)** Schematics of the models used to study exon 10 loss: MO induced alternative splicing (i9 and e10) and CRISPR deleted exon 10 (Mut). Dotted line indicates exon 10 skipping. **(E)** RT-PCR agarose gels showing *NELFE* mRNA isoform switching and reduced full length (FL) 48 hours after treatment with MOs in HCC cell lines. **(F)** Alignment of the d.ex10 band from i9 MO RT-PCR showing exon 10 skipping. **(G)** Alignment of Huh1 d.ex10 mut genomic DNA (top) and cDNA (bottom) sequencing with reference *NELFE* gene and RNA sequences, respectively.

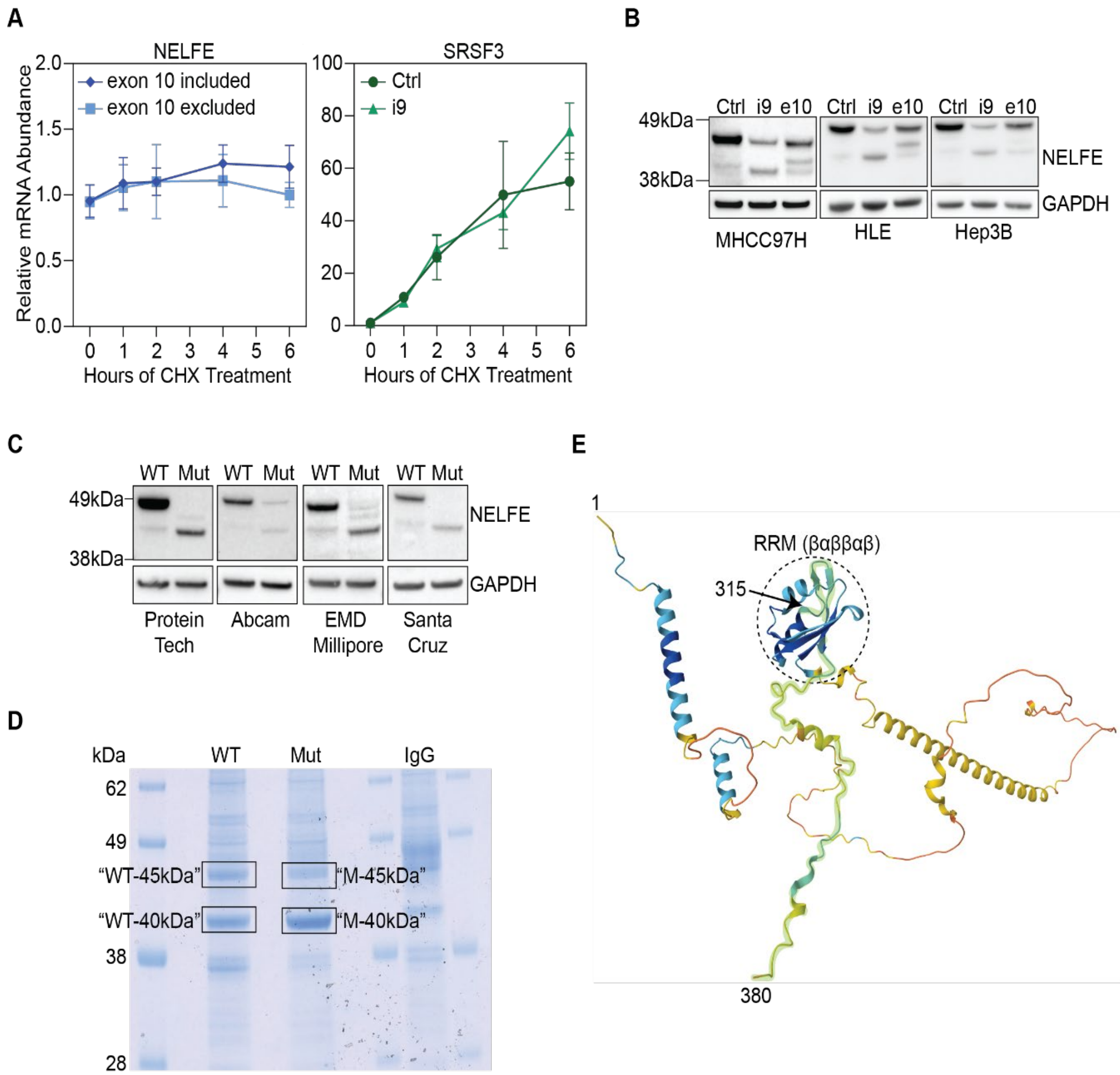

Figure S3. *NELFE* exon 10 skipping produces an uncharacterized protein isoform. (A) RT-qPCR analysis of mRNA abundance after treatment with 0.1mg/mL cycloheximide (CHX) to evaluate if d.ex10 undergoes a translation associated mRNA decay pathway. Left, relative abundance of exon 10 included mRNA from ctrl MO treated cells and exon 10 excluded mRNA from i9 treated cells. Cells were treated with CHX 48 hours after MO transfection. Exon 10 included transcripts were detected using a primer that spans the exon9-exon10 junction. Exon 10 excluded transcripts were detected using a primer that spans the exon9-exon11 junction. Primers detecting the poison exon in SRSF3 were utilized as a positive experimental control. **(B)** Representative immunoblots of NELFE after 72 hours of MO treatment in HCC cell lines. **(C)** Immunoblots of NELFE in Huh1 WT and Huh1 d.ex10 mut cells using multiple NELFE antibodies. **(D)** Representative scan of pre-cut gel submitted for mass spectrometry. Black boxes indicate bands analyzed. Three biological replicates were analyzed. **(E)** AlphaFold prediction for WT NELFE (380aa) folding. The RRM is in the dotted circle. The arrow points to aa315, where the d.ex10 isoform begins to differ and the highlighted region depicts the sequence that differs in d.ex10.

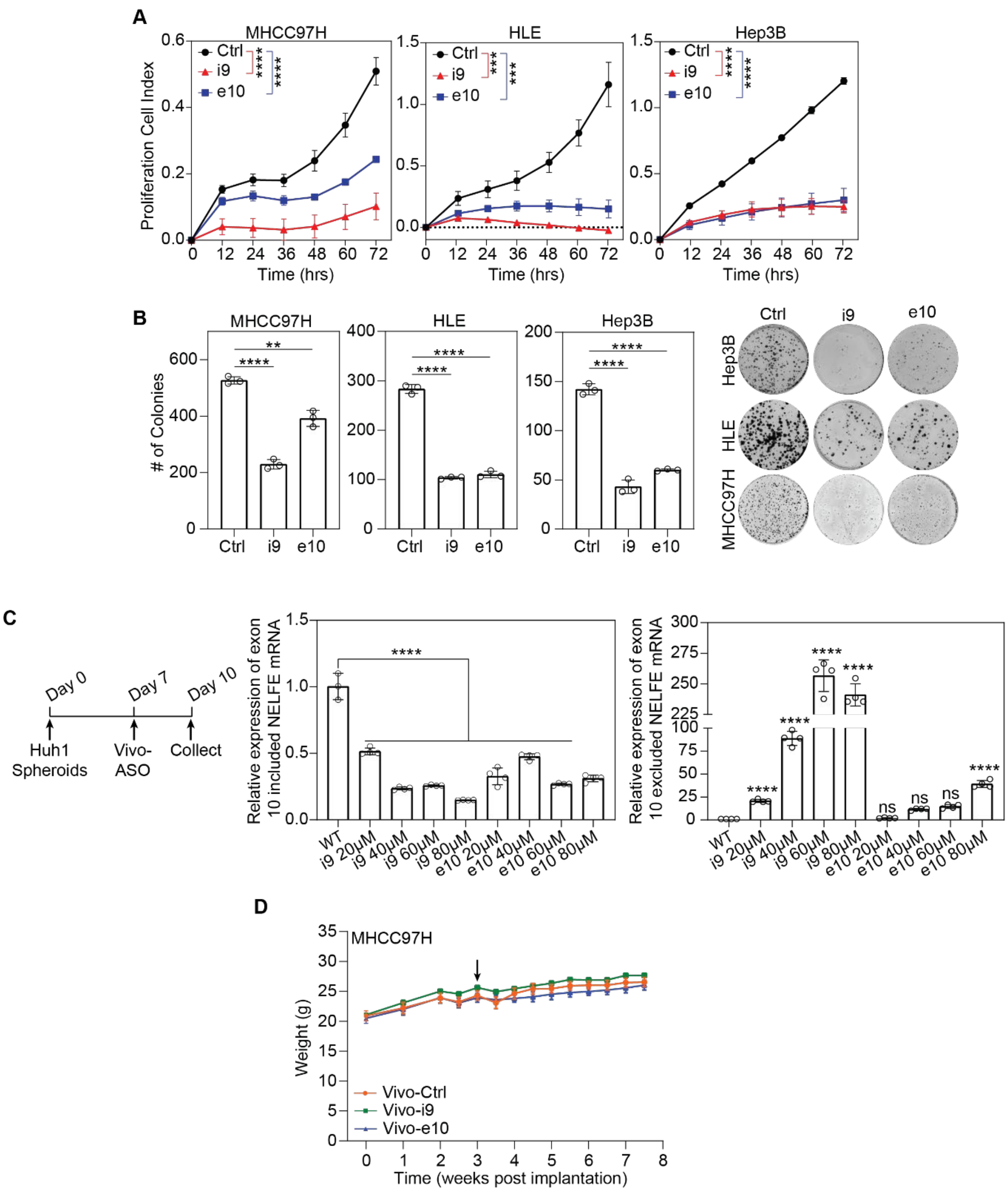

Figure S4. NELFE MOs reduce cancer cell phenotypes across HCC cell lines. (A) Cell proliferation assay in MO treated HCC cell lines. Cells were plated for xCELLigence 48 hours after MO transfection and measured every 12 hours for 72 hours. Data presented as mean± SD (n=3-4). Statistical analysis was conducted at 72 hours with a Student's t-test. ***p<0.001 and ****p<0.0001. **(B)** Cell colony formation assay in MO treated HCC cell lines. Cells were plated 48 hours after MO transfection. Left, quantification of colony formation after 10-14 days. Data

presented as mean ± SD (n=3). Statistical analysis was conducted with a Student's t-test. **p<0.01 and ****p<0.0001. Right, representative crystal violet stains. **(C)** Left, experimental timeline of identifying optimal vivo-MO dosage on spheroids. 150,000 Huh1 WT cells were incubated in an algimatrix for 1 week then treated with vivo-MO for 72 hours. Right, RT-qPCR of exon 10 included and excluded isoforms in the different vivo-MO concentration treated spheroids. Exon 10 included transcripts were detected with a TaqMan probe targeting the exon10-exon 11 junction. Exon 10 excluded transcripts were detected with a TaqMan probe targeting the exon9-exon11 junction. Ct values were normalized to the endogenous control 18S. Data presented as mean ± SD. Statistical analysis was conducted using a one-way ANOVA with Dunnett's multiple comparisons test. ns=not significant and ****p<0.0001. **(D)** Weights of MHCC97H implanted athymic nude mice over the duration of the experiment. Arrow indicates when treatment began. Data are presented as mean ± SEM (vivo-Ctrl n=5, vivo-i9 n=6, vivo-e10 n=5). Statistical analysis was conducted using a two-way ANOVA with Dunnett's multiple comparisons test.

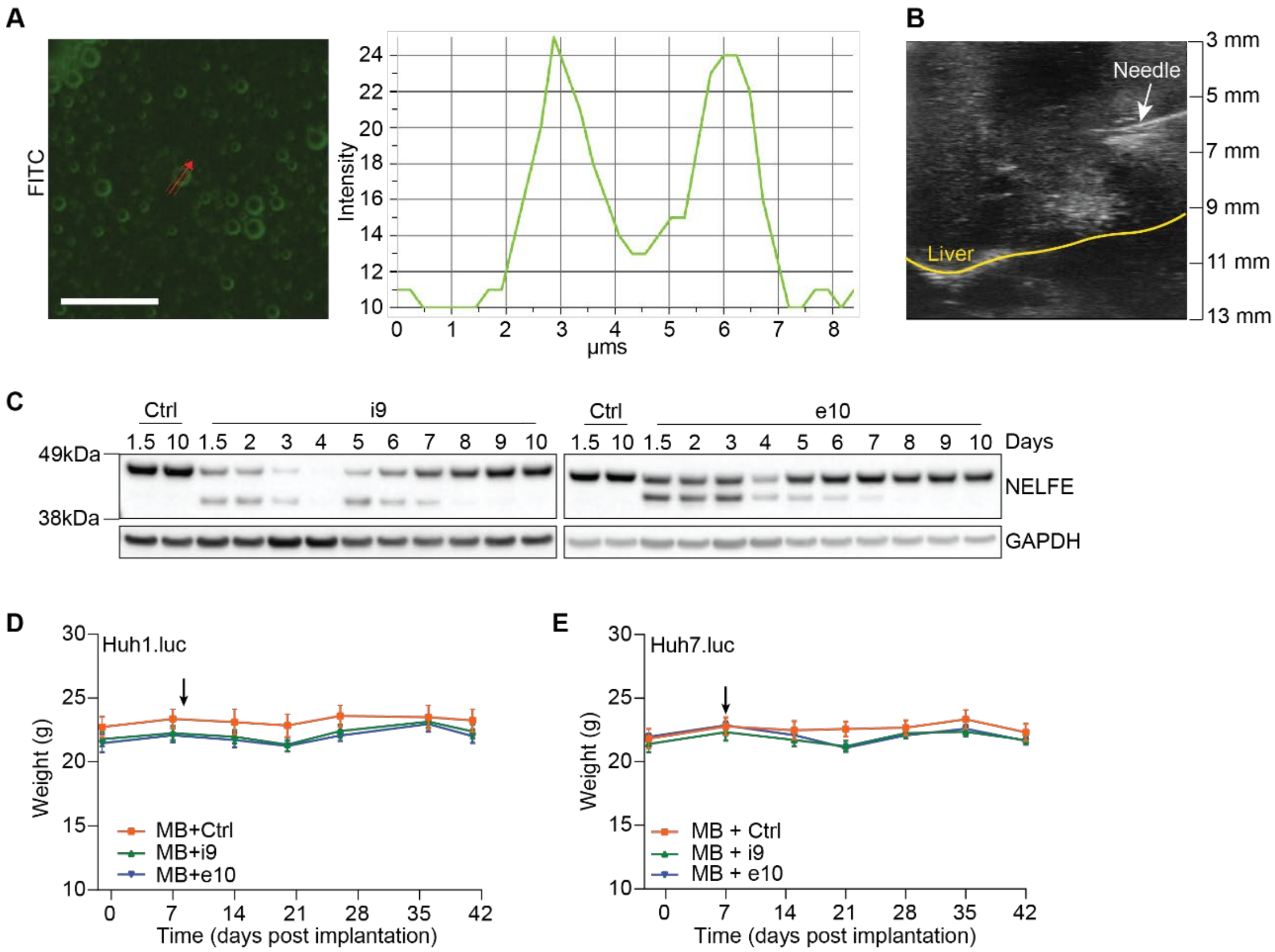

Figure S5. Ultrasound-targeted MBs loaded with NELFE MOs do not affect mice body weight. (A) Quantification of fluorescence across a single MB. Left, a representative FITC image with the red arrow indicating the analyzed section. Scale bar is 20μm. Right, quantification of intensity of fluorescence over analyzed region. The two peaks indicate in increased intensity as bubbles coat the outside. **(B)** Image of ultrasound guided HCC cell injections. **(C)** Immunoblots of NELFE expression over ten days of i9 MO or e10 MO treatment. **(D and E)** Weights of Huh1.luc **(D)** and Huh7.luc **(E)** mice over the treatment weeks of the vivo-MO+MB mice studies. Black arrow indicates when treatment began. Data are presented as mean ± SEM. Statistical analysis was conducted using a two-way ANOVA with Dunnett's multiple comparisons test.

Table S1. Proteomics Results. Mass spectrometry analysis of anti-NELFE immunoprecipitates from Huh1 WT and Huh1 d.ex10 mutant cells, with normal rabbit IgG as the negative control. The complete dataset is provided as a separate Excel file.

Table S2. Cell Line Characteristics.

Cell Line	Histological Type	Race	Age	Sex	HBV	HCV	rs79208225	*NELFE* Copy Number	p53	TERT
Huh1	HCC	Asian	53	M	Y	N	N	3	p.Arg110Leu	
HLE	HCC	Asian	68	M	N	N	N	3	p.Gly244Ala	c.1-124C>T
Hep3B2.1-7	HCC	African American	8	M	Y	N	N	3	null	
Huh7	HCC	Asian	57	M	N	Y	N	2	p.Tyr220Cys	
MHCC97H	HCC	Asian	39	M	Y	N	N	2	p.Glu51Ter	

Table S3. Primer sequences.

Name	Sequence	Experiments
SNP Seq F	GCCGTTGCTGAGGTTGGA	SNP Genotyping
SNP Seq R	TCGGGCTATGTTGACTTTGA	SNP Genotyping
NELFE SNP F2 Long	AGATCTGCCGCCGCGATCGCTGTGCCTTCGTCACCTATGAAAAG	Minigene RT-PCR
pLenti-c-seq R	GATGCCGTAGCAGAGGGTGGT	Minigene RT-PCR, RIP-qPCR
NELFE SNP F2	GGTCGGATTCATTCCCTGAACG	Isoform Switching RT-PCR, Total NELFE RT-qPCR
NELFE SNP R2	GCCATCCACAAGGTTTTCCTTG	Isoform Switching RT-PCR
Trunct1+2 Seq R	CCATGGAGAGGTCAATGATG	Total NELFE RT-qPCR
GAPDH F	TGCACCACCAACTGCTTAGC	Isoform Switching RT-PCR, Total NELFE RT-qPCR
GAPDH R	GGCATGGACTGTGGTCATGAG	Isoform Switching RT-PCR, Total NELFE RT-qPCR
ex8 F	ACATCATTGACCTCTCCATGGAC	CHX RT-qPCR
ex9ex10 R	TCCCGTTGAGCTCAGCAAC	CHX RT-qPCR
cDNAisoformR2	TGTTCTGGACAGCTCAGCAA	CHX RT-qPCR
SRSF3 F	CGTGTAAGAGTGGAACTGTCG	CHX RT-qPCR
SRSF3 poison R	ATGATGGTGACTCTGCGACGA	CHX RT-qPCR
