# Supplementary material for: Morpholino-mediated splice switching of NELFE suppresses hepatocellular carcinoma cell phenotypes in vitro and in vivo
Source: Mol Ther Nucleic Acids. 2026 Oct 1;37(4):103124. doi: 10.1016/j.omtn.2026.103124 (PMC13694868; doi:10.1016/j.omtn.2026.103124)
Supplement: Supplementary file 2 [file mmc3.pdf]

Molecular Therapy
Nucleic Acids
Original Article

Morpholino-mediated splice switching of NELFE suppresses hepatocellular carcinoma cell phenotypes *in vitro* and *in vivo*

Laura M. Reynolds,[1] Ryan Lamm,[1] Kai Zhang,[1] Anna E. Barry-Wolbers,[1] Alvaro Lucci,[1] Brittany N. Ruiz,[1] Corinne E. Wessner,[2] Trish Nguyen,[1] Kayla M. Hammond,[1] Christoph Eckert,[6,7] Ji-Bin Liu,[2] Pongsakorn Choochuen,[1] Kai Ying Hong,[1] Wei Jiang,[3] Marshonna Forgues,[8] Gaia M. Palazzo,[1] Daniel Lin,[4,5] Adam S. Bodzin,[1] Ashesh P. Shah,[1] Xin Wei Wang,[8,9] Sean P. Martin,[10] Nicolas L. Fawzi,[11] Matthias M. Gaida,[6,7] John R. Eisenbrey,[2] and Hien Dang[1,5]

[1]Department of Surgery, Thomas Jefferson University, Philadelphia, PA 19107, USA; [2]Department of Radiology, Thomas Jefferson University, Philadelphia, PA 19107, USA; [3]Department of Pathology and Genome Medicine, Thomas Jefferson University, Philadelphia, PA 19107, USA; [4]Department of Medical Oncology, Thomas Jefferson University, Philadelphia, PA 19107, USA; [5]Sidney Kimmel Comprehensive Cancer Center, Thomas Jefferson University, Philadelphia, PA 19107, USA; [6]Institute of Pathology, University Medical Center Mainz, JGU-Mainz, 55131 Mainz, Germany; [7]TRON, Translational Oncology at the University Medical Center, JGU-Mainz, 55131 Mainz, Germany; [8]Laboratory of Human Carcinogenesis, National Cancer Institute, Bethesda, MD 20892, USA; [9]Liver Cancer Program, Center for Cancer Research, National Cancer Institute, Bethesda, MD 20892, USA; [10]Department of Surgery, Pennsylvania State University College of Medicine, Hershey, PA 17033, USA; [11]Department of Molecular Biology, Cell Biology, and Biochemistry, Brown University, Providence, RI 02912, USA

Hepatocellular carcinoma (HCC) is among the deadliest cancers worldwide, with rising incidence and few targeted therapies, driving high rates of recurrence and poor survival. Negative elongation factor E (NELFE), an RNA-binding subunit of the NELF complex, acts oncogenically when overexpressed and is associated with worse prognosis in HCC, yet no therapies target it. Here, we identify a rare germline intronic variant in the *NELFE* locus, NM_002904.6:c.943-110C>T (rs79208225) that promotes skipping of *NELFE* exon 10 and identifies patients with a trend toward longer recurrence-free survival and lower NELFE expression in their tumors. Mechanistically, the variant reduces U2AF2 occupancy at intron 9, likely through an indirect effect on splice-site recognition rather than direct disruption of U2AF2 binding. Recognizing exon 10 as a targetable splice event, we designed morpholino (MO) antisense oligonucleotides against its splice junctions that phenocopied the variant, lowering NELFE protein expression and impairing malignant phenotypes across HCC cell lines spanning diverse etiologies and NELFE expression levels. Delivered by ultrasound-targeted microbubble destruction (UTMD) in orthotopic liver tumors, NELFE MOs significantly reduced tumor burden and extended survival. These findings establish a naturally occurring splicing event as the basis for a localized, splice-switching therapeutic strategy for HCC.

INTRODUCTION

Liver cancer ranks among the deadliest malignancies worldwide, with new cases and deaths projected to increase by 55% by 2040.[1,2] Hepatocellular carcinoma (HCC) is the most common subtype, accounting for approximately 80% of cases.[3] Prognosis remains poor, with a 5-year survival of only 22%, which is largely due to frequent late stage diagnoses and HCC being a very heterogenous disease.[4,5] HCC often arises from a chronic liver disease (CLD) background with the most common ones being chronic hepatitis B and hepatitis C virus infection, metabolic dysfunction-associated steatotic liver disease, and alcoholic liver disease.[6] Typically, these conditions induce a state of chronic hepatic inflammation, resulting in progressive fibrosis and cirrhosis through the accumulation of genetic and epigenetic alterations.[7] This global dysregulation, combined with the widespread upregulation of RNA binding proteins (RBPs) in HCC, suggests that RBPs are key mediators of tumorigenesis.[8,9] Among the most significantly dysregulated RBPs in HCC is negative elongation factor E (NELFE).[8]

NELFE is a subunit of the NELF complex, which plays an essential role in transcriptional regulation.[10,11] Specifically, NELFE can bind nascent RNA, facilitate its association with cap-binding complexes, and promote promoter-proximal pausing.[12,13] NELFE has been found to promote tumorigenesis by modulating the stability of oncogenes and tumor suppressors in multiple cancer types, including gastric cancer, pancreatic cancer, and HCC.[8,14–16] Moreover, high

Received 7 April 2026; accepted 30 September 2026;
https://doi.org/10.1016/j.omtn.2026.103124.

Correspondence: Hien Dang, Thomas Jefferson University, Philadelphia, PA, USA.
E-mail: hien.dang@jefferson.edu

© 2026 The Author(s). Published by Elsevier Inc. on behalf of The American Society of Gene and Cell Therapy.
This is an open access article under the CC BY license (http://creativecommons.org/licenses/by/4.0/).

expression of NELFE is associated with poor clinical outcomes in these cancers as well as in breast cancer and oral squamous cell carcinoma, and 3%–17% of HCC patients have an increase in *NELFE* gene copy number (Liver HCC, The Cancer Genome Atlas [TCGA], PanCancer Atlas, cBioPortal).[8,14–18] While there is growing evidence that NELFE may be important for tumorigenesis across many cancer types, there are no targeted therapies against it.

Targeted therapeutic options for HCC remain limited, as only a minority of patients have druggable alterations so genetic screening for therapy guidance is not common.[19] Instead, patients with advanced disease typically receive the combination immunotherapies atezolizumab + bevacizumab or tremelimumab + durvalumab.[20,21] These have improved overall survival (OS) of advanced HCC patients from approximately 13–14 months to 16–19 months.[20] However, many patients still fail to respond, develop resistance, or recur.[22] Considering HCC often arises from cirrhosis, treatment options are more limited for those with hepatic dysfunction.[23] Therefore, there remains an urgent need for more efficacious targeted therapies for HCC patients.

Ultrasound-targeted microbubble destruction (UTMD) is an emerging method for localized drug delivery.[24] Microbubbles (MBs) are ultrasound contrast agents that can be loaded with molecules, such as antisense oligonucleotides (ASOs), and selectively released within a tumor through UTMD.[24] UTMD causes sonoporation resulting in temporary cell membrane permeabilization, cytoskeletal rearrangements, and enhanced transmembrane transport, thereby increasing molecule uptake.[25] Preclinical work has demonstrated that UTMD mediated delivery of drug- or gene-loaded MBs improves therapeutic efficacy for liver diseases including liver cancer.[26] ASOs are short, single stranded molecules that bind to complementary RNA sequences to modulate their processing and expression.[27] Specifically, ASOs can sterically hinder spliceosome formation to induce alternative splicing.[28] Vivo-morpholino (MO) ASOs have a covalently linked delivery dendrimer, which enhances endosomal escape and may facilitate direct penetration of the plasma membrane.[29,30] Together, these features suggest that targeted delivery of vivo-MO via UTMD could reduce the required effective concentrations, which is particularly important for the many HCC patients with liver dysfunction.[23]

Here, we describe a therapeutic avenue for HCC by targeting the RBP NELFE via splice modulating MOs. Notably, we identified a previously uncharacterized germline intronic single nucleotide polymorphism (SNP), NM_002904.6:c.943-110C>T (rs79208225). Minigene assays show the SNP promotes alternative splicing of *NELFE* exon 10. MOs induced this alternative splicing event through steric blockade of the exon 10 splice junctions, reducing NELFE expression and cancer-associated phenotypes *in vitro*, while extending survival *in vivo* when delivered via UTMD. This approach was effective across HCC cell lines of diverse etiologies and varying NELFE expression levels, highlighting its potential as a therapeutic strategy for a disease with limited targeted options.

RESULTS

Rs79208225 SNP in HCC patients is associated with a trend toward improved RFS and decreased NELFE expression

To identify mutations affecting NELFE, we performed Sanger sequencing (Sanger-seq) on 81 resected tumors (Figure S1A). We found no somatic mutations but detected a germline heterozygous variant, NM_002904.6:c.943-110C>T (rs79208225), in the *NELFE* locus (Figure 1A). Because *NELFE* is encoded on the minus strand, this variant corresponds to a C>T transition within intron 9. The SNP has an allele frequency of less than 1% in the general population (gnomAD). In our HCC cohort, patients carrying the SNP trended toward longer 5-year recurrence-free survival (RFS) than wild-type (WT) patients (median RFS unreached vs 24 months, $p = 0.064$), whereas 5-year OS did not differ (median OS not reached in either group, $p = 0.35$) (Figures 1B and S1B). We next examined *NELFE* mRNA. Both WT and SNP patients expressed higher *NELFE* mRNA in tumor than in matched non-tumor tissue (WT $p < 0.0001$, SNP $p = 0.007$, Figure S1C). Within tumor tissue, however, *NELFE* mRNA was lower in SNP than in WT patients ($p = 0.04$), while non-tumor levels were comparable between groups ($p = 0.23$) (Figure 1C). Quantification of NELFE staining (percent positive cells) across tumors showed a lower proportion in SNP than WT cases, though the difference was not statistically significant ($p = 0.23$, Figure 1D).

Rs79208225 SNP promotes alternative splicing of *NELFE* exon 10 producing an uncharacterized *NELFE* mRNA isoform

To assess how the rs79208225 SNP affects *NELFE* splicing, we generated minigene reporters spanning exons 9–11 and their intervening introns, fused to green fluorescent protein (GFP) and carrying either the WT or SNP allele (Figure 2A, top). By reverse-transcription PCR (RT-PCR), the SNP reporter produced an additional, smaller band confirmed by Sanger-seq as exon 10 skipping (Figure 2A, bottom, Figure S2A). Because exon 10 is 103 nucleotides, its loss frameshifts exon 11 and shifts the stop codon downstream into the former 3′ UTR. To quantify splicing efficiency, we used a dual-luciferase reporter in which the *NELFE* genomic region was placed immediately upstream of *Renilla* luciferase, with *firefly* luciferase driven by a separate promoter as the transfection control (Figure 2B, top). Exon 10 inclusion preserves the *Renilla* reading frame, whereas skipping introduces a premature stop codon that lowers *Renilla* output in proportion to the degree of skipping. The SNP allele increased exon 10 skipping relative to WT (Figure 2B, bottom). Full-length (FL) transcript (ex9-ex10-ex11) nonetheless remained detectable in both assays, indicating that the SNP shifts splicing in only a subset of transcripts rather than abolishing exon 10 inclusion. Consistent with the reporter data, the exon 10 skipped transcript was present in SNP patient tumors but absent from WT tumors (Figures 2C and S2B).

To investigate the mechanism driving exon 10 skipping, we used RPISeq, a sequence-based predictor of RNA-protein interactions, to score candidate splicing regulators against a 41-nucleotide window centered on the SNP (20 nucleotides on each side). RPISeq

applies two machine-learning classifiers, a support vector machine (SVM) and a random forest (RF), trained on non-redundant RNA-protein interaction datasets to predict binding from sequence alone.[31,32] All six regulators examined, U2AF2, hnRNPUL1, RBM5, hnRNPL, SRSF3, and PTBP1,[33] scored as positive interactors under the SVM classifier (Figure 2D), and U2AF2 showed the highest probability under the RF classifier, which outperforms the SVM in benchmarking.[32] Consistent with these predictions, cross-linking and immunoprecipitation (CLIP, CLIP-seq) data from HepG2 cells in ENCODE placed the SNP directly within the U2AF2 binding region, with RBM5 and SRSF3 sites in the adjacent sequence (Figure S2C). U2AF2 is among the first factors to engage introns during pre-spliceosome assembly,[34] while RBM5 and SRSF3 are established regulators of alternative splicing.[35]

Because the SNP falls within the U2AF2 binding region, had the highest predicted interaction confidence, and is an early essential splicing protein, we examined whether the variant alters U2AF2 engagement at intron 9. Using RIP-qPCR in cells transfected with the WT or SNP minigene reporters, which retain full intronic sequence and generate pre-mRNA substrates spanning the U2AF2 binding context, we found reduced U2AF2 occupancy on the SNP substrate compared to WT (Figure 2E). However, the variant is a pyrimidine-preserving change located approximately 110 nucleotides upstream of the exon 10 3′ splice site, so the observed reduction is unlikely to reflect direct disruption of U2AF2 binding.

To test the functional consequences of exon 10 loss, we designed two MOs targeting either the intron 9-exon 10 (i9) or exon 10-intron 10 (e10) splice junction to pharmacologically induce exon 10 skipping (Figure S2D). Across five HCC cell lines, both MOs reduced total NELFE levels and induced exon 10 skipping relative to scramble control (ctrl) MO (Figures 2F–2H, S2E, and S2F). We refer to this uncharacterized exon 10-skipped isoform as d.ex10. To genetically validate the MO phenotype, we used CRISPR-Cas9 with paired guide RNAs flanking exon 10 to generate a deletion model through non-homologous end joining (Figure S2D), isolating a single clone in Huh1 cells (termed mutant [mut]) confirmed by Sanger-seq of genomic DNA (gDNA) and cDNA. TA cloning of the amplicons followed by sequencing of individual clones confirmed that the deletion was homozygous (Figure 2I, top, Figure S2G). Consistent with the MO data, the mutant showed significantly reduced total *NELFE* mRNA relative to WT, though less markedly than MO-treated cells (Figure 2I, bottom).

D.ex10 mRNA isoform has a similar metabolism as FL mRNA transcripts and produces an uncharacterized NELFE protein isoform

Exon 10 skipping results in a frameshift that causes read through of the normal stop codon, extending translation 61 nt into the former 3′ UTR before terminating at a downstream stop codon, generating d.ex10 isoforms with 3′ UTRs distinct from WT (Figure 3A). Because 3′ UTRs play a central role in post-transcriptional control by regulating transcript stability, translation, and localization,[36] we asked how these altered sequences affect d.ex10 expression. We first tested whether d.ex10 is subjected to translation-associated mRNA surveillance such as nonsense-mediated decay (NMD). Because the reading frame extends to a stop codon in the terminal exon downstream of the normal stop, d.ex10 is not predicted to be an NMD substrate. Consistent with this, cycloheximide (CHX), which blocks translation and its associated decay pathways, did not alter d.ex10 levels while stabilizing a control NMD substrate, indicating that d.ex10 does not undergo translation-associated decay (Figure S3A). Transcript half-life following actinomycin D (act. D) or 5,6-dichlorobenzimidazole 1-β-D-ribofuranoside (DRB) treatment was likewise comparable between the two isoforms (Figures 3B and 3C). Despite this similar stability, both the longer and shorter fragments of the d.ex10 3′ UTR reduced *Renilla* luciferase output relative to the WT 3′ UTR (Figure 3D).

Consistent with the reduced *NELFE* mRNA in MO-treated cells, NELFE protein expression was reduced across MO-treated HCC cell lines (Figures 3E and S3B). Loss of NELFE also destabilized the broader NELF complex, lowering expression of the remaining subunits (Figure 3E), consistent with prior reports.[37,38] A comparable reduction across all NELF subunits was observed in the d.ex10 mutant cells (Figure 3F). In both the models, we detected a lower-molecular-weight band. To determine whether this band was NELFE-derived, we probed it with multiple NELFE antibodies raised against distinct regions of the protein, all of which recognized it (Figure S3C). A small interfering RNA (siRNA) targeting the *NELFE* exon 10 region failed to affect this band, whereas an siRNA targeting *NELFE* exon 4 eliminated it entirely (Figure 3G). Mass spectrometry of the truncated isoform further confirmed the d.ex10 protein (Figure S3D; Table S1). Consistent with a truncated product, the 61 nt read through does not offset the 103 nt lost through exon 10 skipping, yielding a net shorter coding sequence and the lower-molecular-weight species observed by immunoblot (Figure 3E). Because the frameshift is predicted to remove the C-terminal portion of the NELFE RRM, a region that undergoes major conformational rearrangement upon RNA binding, the d.ex10 isoform is likely to have altered RNA-binding capacity (Figure S3E).[39] Importantly, expression of the d.ex10 isoform tagged with GFP in a stable shNELFE cell line resulted in similar cell proliferation rates to NELFE knockdown cells, whereas full length NELFE tagged with GFP was able to partially rescue cell proliferation compared to control cells (Figure 3H), indicating the d.ex10 protein is functionally impaired.

MO induced *NELFE* exon 10 skipping reduces cancer cell phenotypes

Despite growing evidence supporting NELFE's role in HCC and other cancers, no targeted therapies against it exist.[8,14–16,18] Because genetic loss of NELFE attenuates cancer-associated phenotypes,[8,40] we asked whether NELFE MOs could phenocopy this effect pharmacologically. NELFE MOs impaired cell proliferation, colony formation, and 3D spheroid formation, with surviving spheroids appearing smaller than ctrl MO-treated spheroids

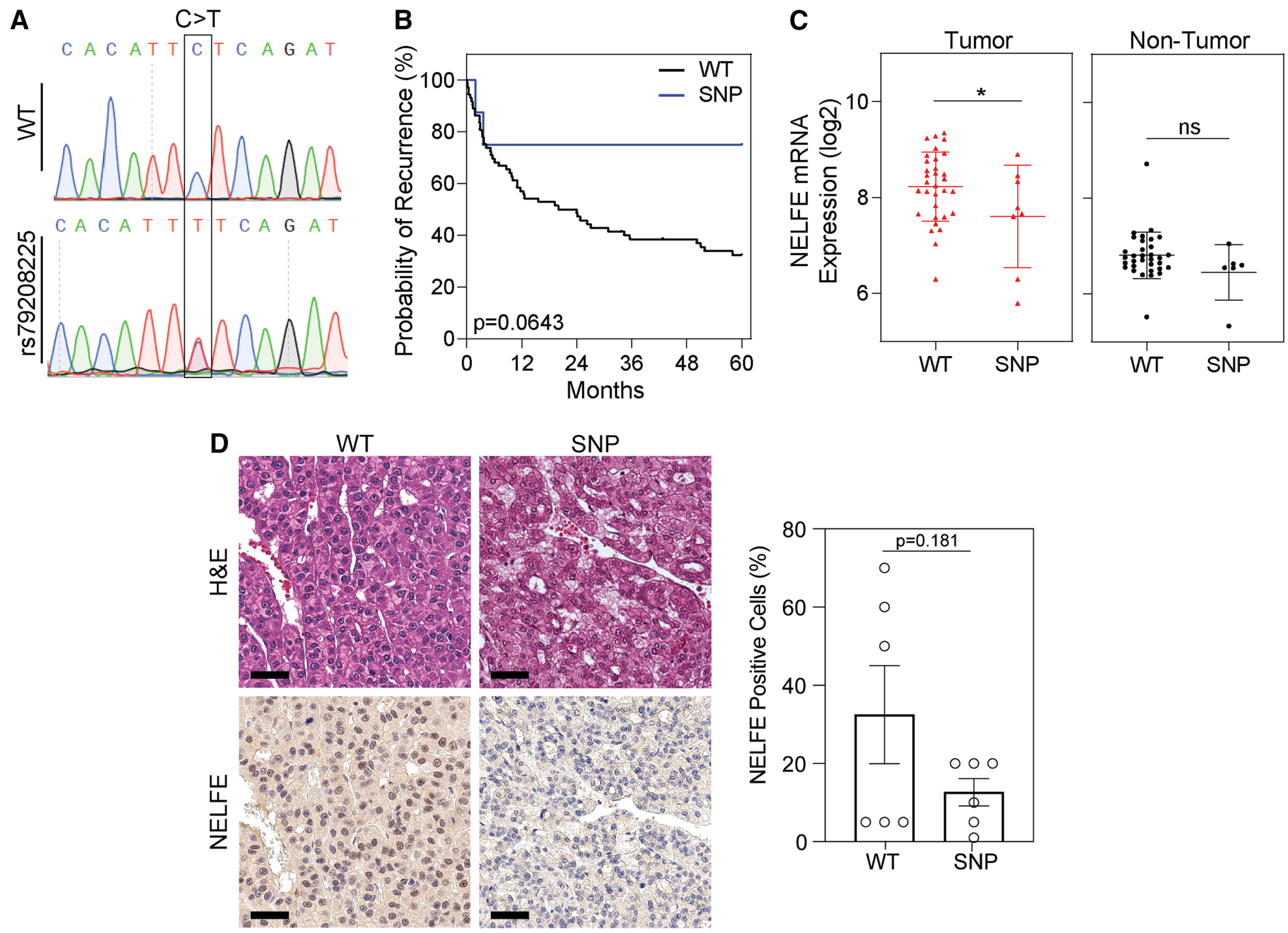

Figure 1. Rs79208225 SNP in HCC patients is associated with improved recurrence-free survival and decreased NELFE expression

(A) Snapshot of Sanger sequencing from a wild type (WT) and a heterozygous NM_002904.6:c.943-110C>T (rs79208225) variant carrying patient. (B) Kaplan-Meier 5 year recurrence-free survival curve for WT and rs79208225 SNP patients (WT n = 73, SNP n = 8, log rank test). (C) TaqMan RT-qPCR to evaluate *NELFE* mRNA expression in WT and rs79208225 SNP patient tumor (WT n = 32, SNP n = 8) and non-tumor (WT n = 33, SNP n = 6) tissue. Data are presented as mean ± SD. Student's t test, *p <0.05. (D) Representative images of hematoxylin & eosin and corresponding NELFE immunohistochemistry on WT or rs79208225 SNP patient tumors. Scale bars, 50 μm (WT n = 6, SNP n = 6). Data presented as mean ± SEM. p value from Welch's test.

(Figures 4A–4C, S4A, and S4B). Consistently, d.ex10 mutant cells showed reduced proliferation and spheroid formation alongside reduced NELF protein expression compared to WT cells (Figures 4D and 4E) across HCC lines with diverse disease etiologies, mutational profiles, and NELFE expression levels (Table S2). To assess NELFE MO efficacy *in vivo*, we first confirmed that NELFE vivo-MOs, MOs conjugated to a triazine-core cell-penetrating dendrimer bearing eight guanidine head groups, induced d.ex10 in a dose-dependent manner in HCC spheroids (Figure S4C).[41] We then tested vivo-MO efficacy in subcutaneous (s.c.) MHCC97H xenografts. Once tumors reached approximately 100 mm, mice received intraperitoneal (i.p.) injections of control or NELFE vivo-MOs (i9 or e10) every other day until ethical endpoints. At 7.5 weeks, NELFE vivo-MO-treated tumors showed substantially reduced tumor burden relative to controls (Figure 4F). Consistent with on-target activity, NELFE vivo-MO-treated mice had lower tumor weights, reduced total *NELFE* mRNA and protein, and enriched d.ex10 mRNA expression, with no change in body weight (Figures 4G–4I and S4D).

Ultrasound-targeted destruction of NELFE MO-loaded MBs extends survival *in vivo*

The NELF complex is an essential transcription regulator in normal cells; therefore, tumor-localized delivery of NELFE MOs is a prerequisite for clinical translation.[42,43] MBs are currently used clinically for contrast-enhanced ultrasound imaging and are in clinical trials alongside conventional cancer therapies.[44] UTMD drives sonoporation, transient membrane permeabilization at the site of bubble cavitation that enhances local drug uptake.[25] This approach has shown clinical benefit in HCC as patients receiving Y-TARE with UTMD had higher rates of partial or complete response and longer OS than those receiving Y-TARE alone.[45] Preclinical studies have similarly demonstrated improved drug transfection efficiencies in HCC models with UTMD compared to no UTMD or drug administration alone.[46–48] On this basis, vivo-MOs were loaded onto Definity MBs through charge-charge interactions during MB activation for targeted delivery via UTMD (Figures 5A–5C and S5A). To evaluate whether UTMD improves delivery of NELFE vivo-MOs, we treated Huh1 spheroids with an untreated control and low and high MO

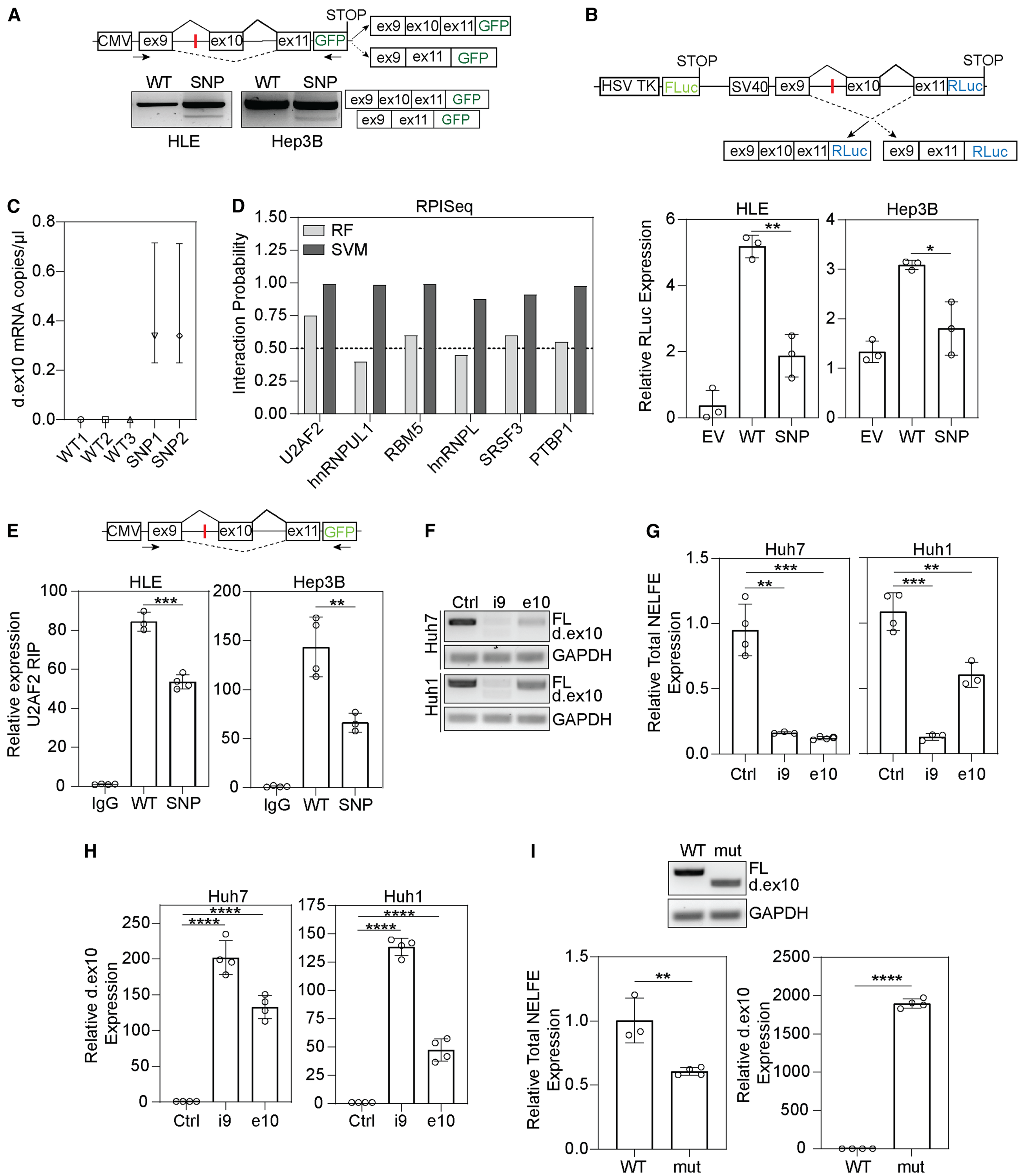

Figure 2. Rs79208225 SNP promotes alternative splicing of *NELFE* exon 10 producing an uncharacterized *NELFE* mRNA isoform

(A) Top, schematic of the minigene reporter. SNP minigenes have the rs79208225 C>T variant at the red line. WT minigenes retain the C allele. Arrows indicate primers for RT-PCR. Dotted line represents exon 10 skipping. Bottom, RT-PCR agarose gel of WT or rs79208225 SNP minigene transfected HCC cells. Rs79208225 SNP minigene cells contain a smaller band. CMV, cytomegalovirus; ex, exon; GFP, green fluorescent protein. (B) Top, schematic of the psiCheck dual luciferase firefly (fluc) and *Renilla* (Rluc)

(legend continued on next page)

concentrations of vivo-i9 alone, vivo-i9 with MBs, and vivo-i9 with MBs plus UTMD. NELFE knockdown was greatest with vivo-i9 plus MBs plus UTMD at the high concentration (Figure 5D).

To test this approach *in vivo*, luciferase-expressing HCC cells (Huh1.luc and Huh7.luc) were implanted into the livers of non-obese diabetic/severe combined immunodeficiency (NOD/SCID) mice under ultrasound guidance (Figure S5B). Seven days after implantation, tumors were confirmed by bioluminescence imaging. Mice were randomized and treated retro-orbitally with vivo-MO-loaded MBs twice a week, based on optimal time efficacy *in vitro*, for a total of six doses (Figure S5C). NELFE vivo-MO-loaded MBs delivered by UTMD (MB-i9 and MB-e10) attenuated tumor growth relative to UTMD with control MBs (UTMD+MB-Ctrl) in both models (Figures 5E and 5F) and conferred a modest survival benefit. Median survival was 44 days for MB-Ctrl, 58 days for MB-i9, and 61 days for MB-e10 in Huh1.luc-bearing mice (p = 0.0006, Figure 5E), and 49 days for MB-Ctrl, 65 days for MB-i9, and 66.5 days for MB-e10 in Huh7.luc-bearing mice (p < 0.0001, Figure 5F). Body weight did not differ across groups in either model, indicating that localized delivery was well-tolerated without overt systemic toxicity (Figures S5D and S5E).

DISCUSSION

HCC remains a highly lethal malignancy with limited treatment options, underscoring the urgent need for innovative therapeutic strategies. Elevated expression of NELFE is associated with disease progression and poor clinical outcomes in HCC patients.[8,40] Here, a rare germline SNP (rs79208225) that promotes *NELFE* exon 10 skipping led us to develop MOs that pharmacologically phenocopy the rs79208225 splicing event through steric blockade of exon 10 splice junctions, lowering NELFE and NELF protein expression and impairing cancer cell phenotypes *in vitro* and *in vivo*. Delivered by UTMD, these splice-switching MOs attenuated tumor growth and modestly extended survival in orthotopic HCC models, establishing a framework for localized, tumor-restricted targeting of an otherwise essential transcriptional regulator.

rs79208225 has not been characterized in HCC, and patients carrying it trended toward improved RFS, but given its low population frequency (under 1% generally and approximately 2.5% in Asian populations; gnomAD v.4.1.0), its enrichment in our cohort, and the modest effect size, we do not propose it as a prognostic biomarker; rather, it served as a template identifying exon 10 as an actionable splice target. Mechanistically, the variant lies deep within intron 9, is pyrimidine-preserving, and reduces U2AF2 occupancy likely without directly disrupting U2AF2 binding. We therefore favor an indirect effect in which an adjacent factor, most likely RBM5, modulates U2AF2 recruitment. Exon 10 skipping generates a previously uncharacterized transcript, d.ex10, which is stably expressed and escapes NMD. The resulting isoform loses the C-terminal RRM, is predicted to have altered RNA-binding and NELF complex function and is unable to rescue HCC cell proliferation. Still, the mechanism driving the reduction in steady-state *NELFE* mRNA, which contributes to the decreased NELFE protein production, remains to be defined. Since NELFE is known to interact with RNA-CBC complexes, its loss can decrease cap stability and increase of occupancy of XRN2, an exonuclease, and DCP2, a decapping enzyme, at transcription start sites. Therefore, it is possible loss of NELFE, or at least the functionally important region for interactions with CBC, results in degradation of its own pre-mRNA.

MOs are established tools for splice modulation, and six of the eleven FDA-approved ASOs act through this mechanism.[49,50] We designed MO ASOs that phenocopy the rs79208225 splicing event through steric blockade of the exon 10 splice junctions, reproducing the effects of genetic NELFE loss and significantly reducing proliferation and impairing colony and spheroid formation across HCC lines of diverse etiology.[8,40] Although most approved ASOs treat neurodegenerative disease, several are in late-stage trials for liver disease, including phase 3 agents for chronic hepatitis B (bepirovirsen), alpha-1 antitrypsin deficiency (fazirsiran), and MASH (AZD2693), etiologies that predispose to HCC.[6,27,51,52] A central barrier for oligonucleotide therapeutics is endosomal entrapment, with only 1%–2% typically reaching the cytosol.[53] The octaguanidinium dendrimer conjugated to our vivo-MOs is designed to enhance endosomal escape and may promote direct membrane penetration, potentially mitigating this limitation.[29,30]

Because the NELF complex is essential for normal transcription and contributes to muscle and intestinal repair,[54,55] systemic NELFE inhibition would risk dose-limiting toxicity in healthy tissue, a concern compounded in HCC patients with underlying CLD.[23] Localized delivery is therefore critical. We observed no weight loss in subcutaneously-treated mice, but this does not establish systemic safety, because the human-sequence MOs are mismatched to murine NELFE and do not engage the target in mouse tissue. To restrict exposure to tumor, we delivered NELFE MOs by UTMD, which

reporter for WT or rs79208225 SNP minigenes. Bottom, cells were transfected with WT or SNP reporter to quantify alternative splicing. Data presented as mean ± SD (n = 3). Student's t test, *p <0.05; **p <0.01. EV, empty vector control. (C) Digital PCR graph of d.ex10 mRNA isoform in HCC tumors from patients that harbor the rs79208225 SNP. Data presented as concentration (copies/μL) ± 95% confidence intervals. (D) RPIseq scores of the interaction probability of different splicing related proteins and the 41 nt sequence around the rs79208225 locus (20 nt on each flank) on the minus strand. Probability > 0.5 indicates positive interaction. SVM, support vector machine,; RF, random forest classifier. (E) Top, schematic of minigene reporters with RT-qPCR primers. Bottom, U2AF2 RNA immunoprecipitation PCR analyses of WT or SNP minigene transcripts. Data are presented as mean ± SD (n = 3). Student's t test, **p <0.01 ; ***p <0.001. (F) RT-PCR agarose gel of FL *NELFE* and isoform at 48 h of MO treatment in HCC cell lines. (G) RT-qPCR detection of total *NELFE* mRNA and (H) d.ex10 isoform after 48 h of MO treatment. Total *NELFE* mRNA was evaluated with primers in exon 8. D.ex10 isoform was evaluated with a TaqMan probe for the exon9-exon11 junction. Data are presented as mean ± SD (n = 4). Student's t test, **p <0.01, ***p <0.001. (I) Top, RT-PCR agarose gel of exon 10 deletion CRISPR model (mut) in Huh1 cells. Bottom, RT-qPCR analysis of total *NELFE* mRNA (left) and d.ex10 isoform (right) (n = 4). Student's t test, **p <0.01; ****p <0.0001.

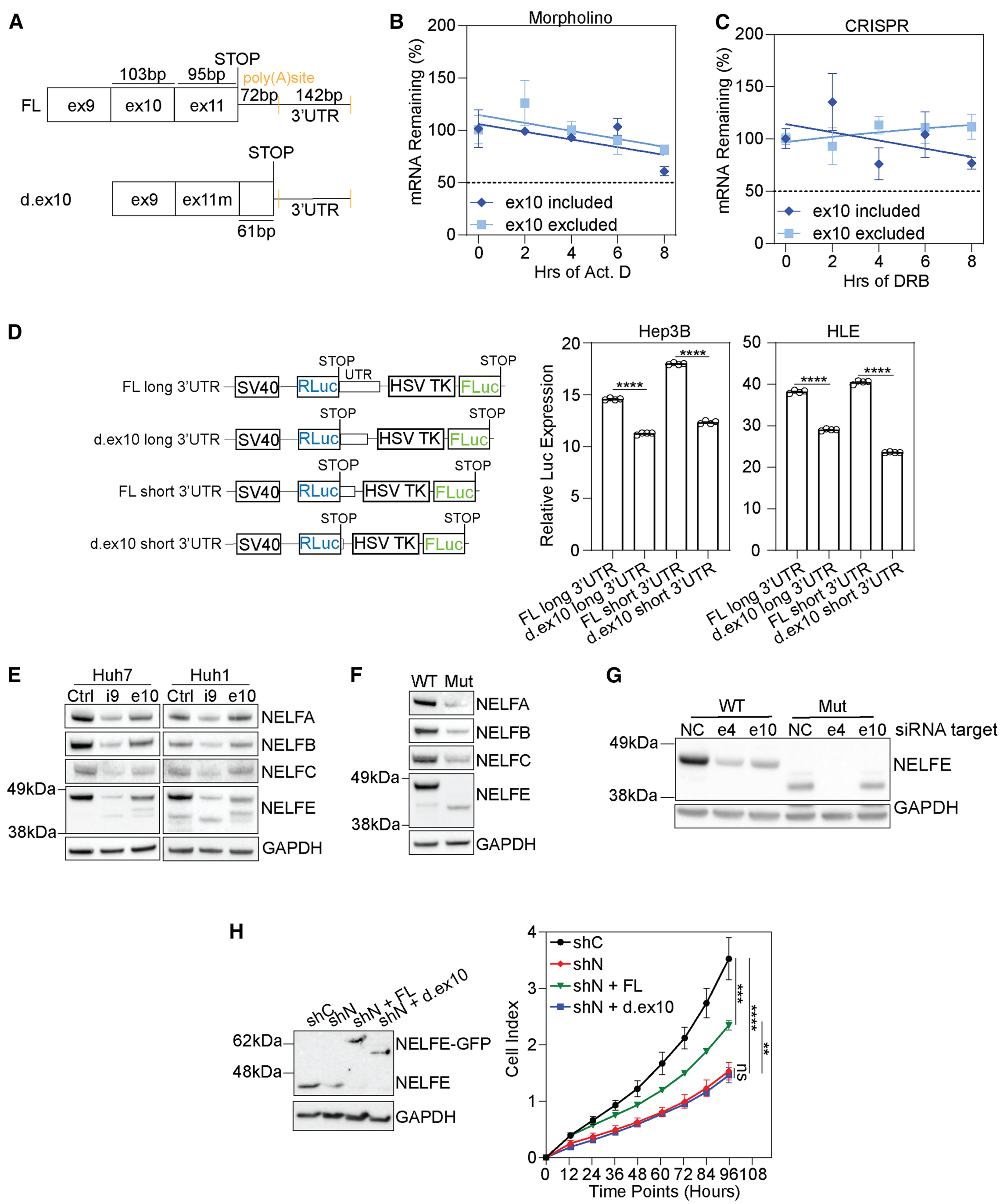

Figure 3. d.ex10 mRNA isoform has a similar metabolism as FL mRNA transcripts and produces an uncharacterized NELFE protein isoform

(A) Schematic of the effect of exon 10 skipping on *NELFE* stop codon location and 3′ UTR length. Depending on which poly(A) site is utilized, d.ex10 may have a 3′ UTR length of either 11 or 153 bp. Ex, exon; FL, full length; UTR, untranslated region. (B) mRNA stability evaluation of FL and d.ex10 mRNA transcripts following 5 μg/mL actinomycin D (Act. D) treatment in MO transfected cells or (C) 60 μM 5,6-Dichlorobenzimidazole 1-β-D-ribofuranoside (DRB) treatment in exon 10 CRISPR deletion model. Exon 10 included transcripts were detected with a TaqMan probe at the exon10-exon11 junction. D.ex10 transcripts were detected with a TaqMan probe at the exon9-exon11 junction. Dotted lines are at 50%. Data are presented as mean ± SD with one-phase decay curves (n = 3 in B). (D) Top, schematics of the 3′ UTR psiCHECK-2 dual luciferase reporters. *NELFE* 3′ UTR sequences were cloned immediately downstream of *Renilla's* (RLuc) stop codon. Bottom, HCC cells were transfected with dual luciferase constructs and relative luciferase expression was measured by normalizing *Renilla* luciferase signal to firefly luciferase signal. Data are presented as mean ± SEM (n = 4). Student's t test, ****p <0.0001. FLuc, firefly luciferase. (E) Representative immunoblots of NELF complex subunits after 72 h of MO treatment. (F) Representative immunoblots

(legend continued on next page)

uses sonoporation to enhance local uptake and benefits from the natural hepatic accumulation of both MBs and MOs.[25,56,57] Loading the MO onto the MB surface couples payload release to the site of acoustic destruction, which favors localized deposition relative to co-administration of free MO and MBs. MBs are already tested clinically alongside chemotherapy, radiation, and transarterial radioembolization.[44,45] Six treatment sessions in mice produced a modest but significant survival benefit in both orthotopic models, providing proof-of-concept that UTMD can deliver splice-switching MOs to HCC. Because every arm received UTMD and vivo-MO-loaded MBs plus UTMD demonstrated the greatest NELFE knockdown *in vitro* compared to vivo-MO alone or MBs without UTMD, we suggest the MO cargo delivered via UTMD drives the prolonged survival rather than UTMD alone. However, direct confirmation of on-target NELFE knockdown in tumor tissue following MB-mediated delivery *in vivo* remains an important next step, as the pharmacodynamics and biodistribution of MO-loaded MBs in orthotopic liver tumor models have not yet been fully characterized. Given the consistent effects of NELFE across cancer types, this strategy may warrant investigation in other NELFE-dysregulated malignancies.[14–18] Together, this work establishes a translational framework for targeting NELFE in HCC through UTMD-delivered splice-switching MOs.

MATERIALS AND METHODS

Human samples

Human biological samples were collected with the approval of the Thomas Jefferson University Hospital (TJUH) Institutional Review Boards (IRB Control #20D.138) and in full compliance with the ethical guidelines of the National Institutes of Health (NIH) for the collection and use of human samples for research. Formalin-fixed, paraffin-embedded (FFPE) HCC tissue was collected from surgical TJUH HCC patients from 2017 to 2020. Fresh HCC tissue and/or patient blood was obtained from surgical TJUH HCC patients from 2020 to 2024. 14 patients were from TJUH and 67 patients were from the liver cancer institute dataset.[58] Recurrence time was determined from date of surgery to date of radiographic evidence of disease.

Patient RNA was isolated from FFPE tissue using the High Pure FFPET RNA Isolation Kit (Roche, 6650775001). Relative *NELFE* mRNA expression was detected using TaqMan Fast Advanced Master Mix (Thermo Fisher Scientific, 4444963) and a TaqMan probe for *NELFE* exons 2 and 3 (Thermo Fisher Scientific, Hs00267952_m1) normalized to the endogenous control, *18S* (Thermo Fisher Scientific, Hs99999901_s1). Digital PCR was performed with 3.5 ng cDNA using a custom TaqMan assay for *NELFE* d.ex10 (Thermo Fisher Scientific, APXG4CC) on the Absolute Q Digital PCR system (Thermo Fisher Scientific).

DNA was isolated in one of three methods: (1) gDNA was isolated using the QIAamp DNA mini kit (QIAGEN, 51304) from peripheral blood mononuclear cells isolated from whole blood. (2) Approximately 10 mg of tumor tissue was homogenized (2.9 m/s, 20 s, 1 cycle) and gDNA was isolated with the QIAamp DNA mini kit. (3) gDNA was isolated from FFPE tissue using the QIAamp DNA FFPE Tissue Kit (QIAGEN, 56404). DNA was amplified and Sanger sequenced (Psomagen) with SNP Seq F and SNP Seq R primers and aligned to the reference using SnapGene software (www.snapgene.com) for SNP Genotyping. Primer sequences are listed in Table S3.

Cell culture

The cell lines Huh1 (JCRB, RRID:CVCL_2956), HLE (JCRB, RRID:CVCL_1281), and Huh7 (JCRB, RRID:CVCL_0336) were cultured in Dulbecco's modified Eagle's medium (DMEM) (Gibco, 11965118) supplemented with 10% characterized fetal bovine serum (cFBS), 1% L-glutamine (LG) (Gibco, 25030-081), and 1% Penicillin-Streptomycin-Glutamine (PS) (Gibco, 10378016). Hep3B cells (ATCC, RRID:CVCL_0326) were cultured in minimum essential medium (MEM) (Gibco, 11090099) supplemented with 10% cFBS, 1% LG, 1% PS, 1% MEM non-essential amino acids (Gibco, 11140-050), and 1% sodium pyruvate (Gibco, 11360-070). MHCC97H cells (RRID:CVCL_4971) were maintained in DMEM medium supplemented with 10% defined fetal bovine serum (FBS), 1% LG, and 1% PS. Cells were used within 15 passages of thawing and maintained at 37°C with 5% CO_2. Cells underwent mycoplasma testing every month using the LookOut Mycoplasma PCR Detection Kit (Sigma-Aldrich, MP0035) and authenticated annually by short tandem repeat using the Promega PowerPlex 16 system.

Animal studies

The animal study protocol (#02259) was approved by the Institutional Animal Care and Use Committee at Thomas Jefferson University. 8- to 10-week-old female NOD/SCID (NOD.CB17-Prkdc^{scid}/NcrCrl, RRID:IMSR_CRL:394) mice were obtained from Charles River Laboratories. 8- to 10-week-old female athymic nude (RRID:IMSR_JAX:002019) mice were obtained from Jackson Laboratory (Wilmington, MA, USA). To generate luciferase expressing cells, pFUGW-Pol2-ffluc2-eGFP virus (RRID:Addgene_71394) was transduced into Huh1 and Huh7 cells which were sorted using BD FACSMelody Cell Sorter (BD Biosciences) collecting the brightest 10% of GFP-expressing cells.

MHCC97H cells were trypan blue counted, and 5 million cells were resuspended in Hanks' balanced salt solution (Thermo Fisher Scientific, 14175095) and Matrigel (Corning Life Sciences, CB40234C) then injected into the right flank of athymic nude mice. Tumors were measured twice a week using a caliper and volume was

of NELF complex subunits in Huh1 WT and d.ex10 mutant (mut) cell lines. (G) Representative immunoblots of NELFE after 72 h of knockdown with either an siRNA targeting exon 4 (e4) or exon 10 (e10) to validate the lower band is a NELFE isoform that lacks exon 10 (d.ex10). (H) Left, representative immunoblot of FL or d.ex10 protein rescues tagged with GFP in stable shNELFE knockdown in Hep3B cells. Right, representative cell proliferation of Hep3B cells to assess effect of d.ex10 on cell phenotypes. Data are presented as mean ± SD (n = 3). Student's t test performed at 96 h. $^{**}p$ <0.01, $^{***}p$ <0.001, $^{****}p$ <0.0001.

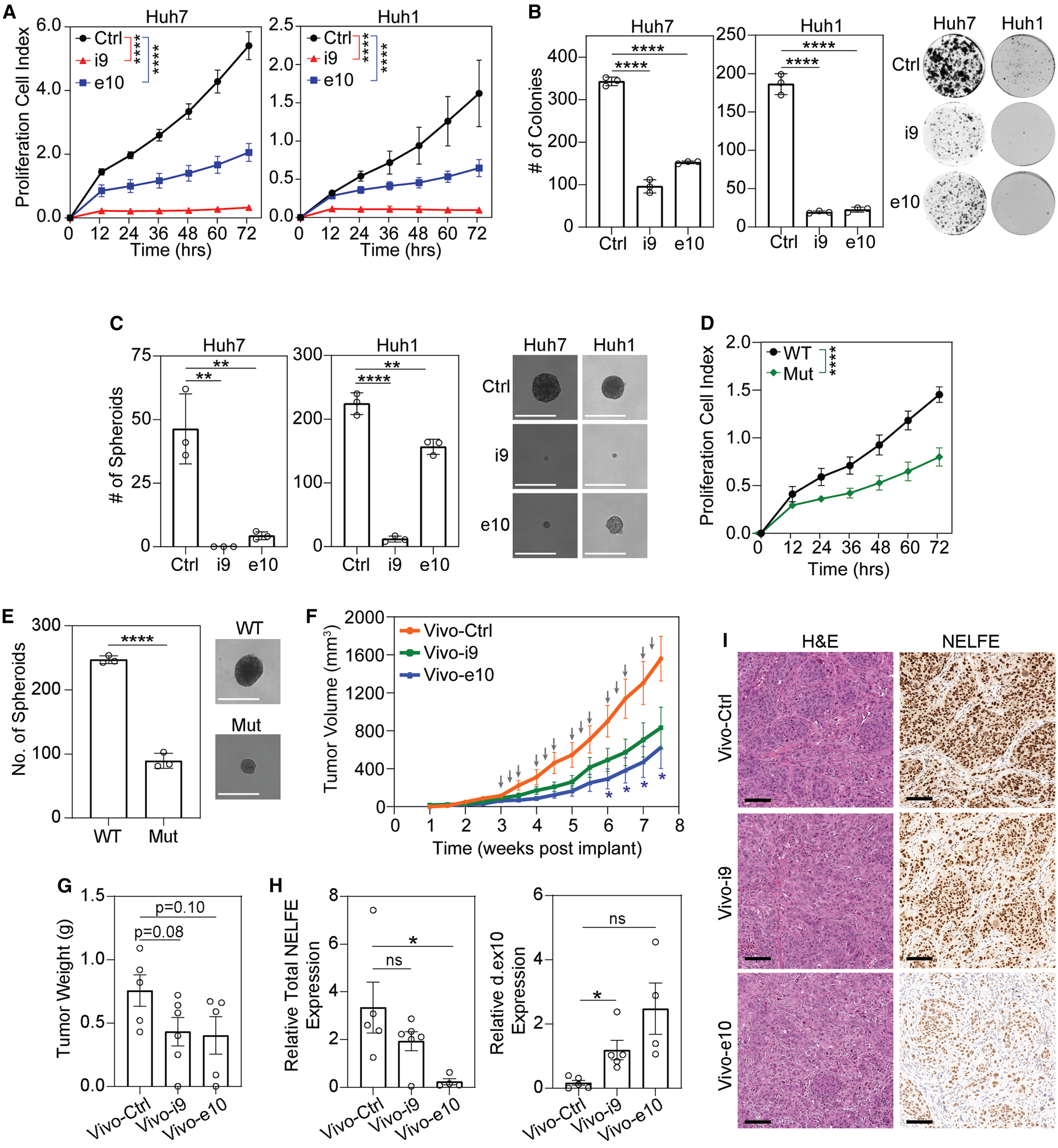

Figure 4. Loss of *NELFE* exon 10 is associated with reduced cancer cell phenotypes

(A) Cell proliferation assay in MO treated HCC cell lines. Cells were plated for xCELLigence 48 h after MO transfection and measured every 12 for 72 h. Data are presented as mean ± SD (n = 3–4). Statistical analysis was conducted at 72 h using a Student's t test. ****p <0.0001. (B) Cell colony formation assay in MO treated HCC cell lines. Cells were plated in a 6-well plate 48 h after MO transfection. Left, quantification of colony formation after 10–14 days. Right, representative crystal violet stains of colonies. Data is presented as mean ± SD (n = 3), Student's t-test. **p <0.01 and ****p <0.0001. (C) Spheroid formation assay in MO treated HCC cell lines. Cells were plated in a 6-well AlgiMatrix 48 h after MO transfection. Left, quantification of spheroid formation after 14 days. Right, representative spheroids imaged at 20×. Scale bars, 200 μm. Data are presented as mean ± SD (n = 3). Statistical analysis was conducted by a Student's t test. (D) Cell proliferation assay in Huh1 WT and Huh1 d.ex10 mut cell lines. Data are presented as mean ± SD (n = 3). Student's t test performed at 72 h. (E) Spheroid formation assay in Huh1 WT and Huh1 d.ex10 mut cell lines. Left, quantification of spheroid

(legend continued on next page)

calculated with the formula: mm = 0.5× (L×W). When tumors reached approximately 100 mm, mice received 80 μM vivo-MO in 50 μL treatments through i.p. injections three times a week until mice reached their ethical endpoints.

For intrahepatic injections, Huh1.luciferase (Huh1.luc) or Huh7.luciferase (Huh7.luc) cells were trypan blue counted, and 1 million cells were resuspended in serum-free DMEM media and Matrigel then injected using ultrasound guided visualization of the NOD/SCID mouse liver. To detect and measure tumor take and growth, mice were given i.p. injections of 30 mg/kg D-Luciferin (Thermo Scientific, 88294) and luciferase signal was captured using the IVIS Lumina III In Vivo Imaging System (PerkinElmer). Images were generated using Aura imaging software (Spectral). Approximately seven days post cell injections, mice with luciferase signal were randomized into treatment groups: vivo-MO standard control (5′-CCTCTTACCTCAGTTACAATTTATA-3′, GeneTools, LLC) loaded MB (MB + Ctrl), i9 vivo-MO loaded MB (MB + i9), or e10 vivo-MO loaded MB (MB + e10). MOs were loaded onto Definity (Lantheus Medical Imaging) MB during activation through charge-charge interactions. Mice were given 80 μM MO in 6 × 10 MBs via retroorbital injection of 50 μL total volume twice per week for three weeks. Assuming a mouse blood volume of 2 mL, this would correspond to a human dosage of roughly 125 mL.[59] This value is considerably higher than current recommendations for diagnostic contrast enhanced ultrasound (recommended infusion of up to 1.3 mL for Definity).[60] While this usage is a different indication compared to current diagnostic approvals, future work should focus on optimizing of MO loading efficiency to better align scaled doses to existing Definity safety data. Ultrasound imaging was performed using dual B-mode (for image navigation) and Cadence Pulse Sequencing (for MB visualization) using an Acuson Sequoia scanner (Siemens Healthineers) with a 10L4 transducer imaging at 18 frames per second. Acoustic characterization was performed in a scanning tank (Onda Aims III Scanning Tank) with 0.5 mm needle hydrophone (Precision Acoustics) in degassed water and found to be transmitting 4.6 MHz pulses with a pulse length of 0.6 μs and peak negative pressures of approximately 0.2 and 2.0 MPa for the low and high mechanical index (MI) settings at the focus. Following MB arrival in the liver, a 4 s destructive pulse was employed to destroy MBs within the tumor (mechanical index = 1.4), followed by approximately 5 s of low MI imaging to visualize reperfusion (mechanical index = 0.12). During the 4 s UTMD, the sonographer would manually sweep to ensure MB destruction throughout the tumor. Immediately post the 4-s UTMD, there was a noticeable decrease in MB signal corresponding to MB destruction. This process was continually repeated, with UTMD lasting approximately three minutes until MBs were no longer visualized. Mice were euthanized at their ethical endpoint to track OS.

In silico protein-RNA interaction prediction

Enhanced cross-linking and immunoprecipitation (eCLIP) sequencing data for target genes were obtained from the ENCODE portal (https://www.encodeproject.org/; accessed October 27, 2025). To generate representative signal tracks, bigWig files from two biological replicates per gene were merged using bigWigMerge producing a single BedGraph file. This BedGraph signal was converted back to bigWig format via bedGraphToBigWig for downstream visualization. Final consolidated tracks were visualized using the integrative genomics viewer to examine proximity of binding regions to the rs79208225 locus.

To further evaluate the potential protein binding at the rs79208225 locus, we employed the RNA-Protein Interaction Prediction (RPI-seq) web server. A 41 nt RNA sequence comprising the rs79208225 locus flanked by 20 nucleotides on both the 5′ and 3′ ends was tested against a panel of splicing related proteins. Interaction probabilities were calculated using the RF and SVM classifiers. An interaction probability >0.5 was considered a positive prediction of binding, with higher scores indicating increased confidence in the physical interaction.

Protein structure prediction

The predicted structure of full-length human *NELFE* (UniProt P18615, 380 amino acids) was retrieved from the AlphaFold Protein Structure Database (v.6, released October 21, 2025; https://alphafold.ebi.ac.uk/entry/P18615).[61] Structures in the database are generated with AlphaFold2. The RRM and the region that differs in the d.ex10 isoform, beginning at amino acid 315, are annotated in Figure S3E.

MO ASO transfections

MOs were designed and manufactured by GeneTools, LLC to target exon 10 splice junctions: I9 (5′-TCCCGTTGAGCTGAAGCAGAAGAGG-3′) targets intron 9-exon 10 and E10 (5′-GAATCCCATTCTTACCGAGGGAGCC-3′) targets exon 10-intron 10. A random control oligo (5′-25*N*-3′) was used as a negative control. MOs were resuspended in UltraPure DNase/RNase free distilled water (Invitrogen, 10977015) for a 1 mM stock concentration. 3.5 μM MO was electroporated in 0.5–1.0 million cells

formation after 14 days. ****p <0.0001. Right, representative spheroids imaged at 20×. Scale bars, 200 μm. (F) MHCC97H tumor growth curve in athymic nude mice treated with intraperitoneal injections of 80 μM vivo-MOs. Gray arrows indicate treatment. Data presented as mean ± SEM (vivo-Ctrl n = 5, vivo-i9 n = 6, vivo-e10 n = 5). Two-way ANOVA with Dunnett's multiple comparisons, *p <0.05. (G) Graph representing tumor weights for indicated groups at the conclusion of the study. Data presented as mean ± SEM (vivo-Ctrl n = 5, vivo-i9 n = 6, vivo-e10 n = 5). Statistical analysis was conducted using a Student's t test. (H) Left, RT-qPCR using a TaqMan probe spanning the exon 2-exon 3 junction to detect total *NELFE* mRNA from a less than 50 mg section of tumor tissue. Right, RT-qPCR using a TaqMan probe spanning the exon 9-exon 11 junction to detect d.ex10 mRNA transcripts. Data are presented as mean ± SEM (vivo-Ctrl n = 5, vivo-i9 n = 6, vivo-e10 n = 4). Student's t test with Welch's correction, *p <0.05 (I) Representative hematoxylin and eosin (H&E) stain and NELFE immunohistochemistry of MHCC97H tumors with different vivo-MO treatments. Scale bars, 100 μm.

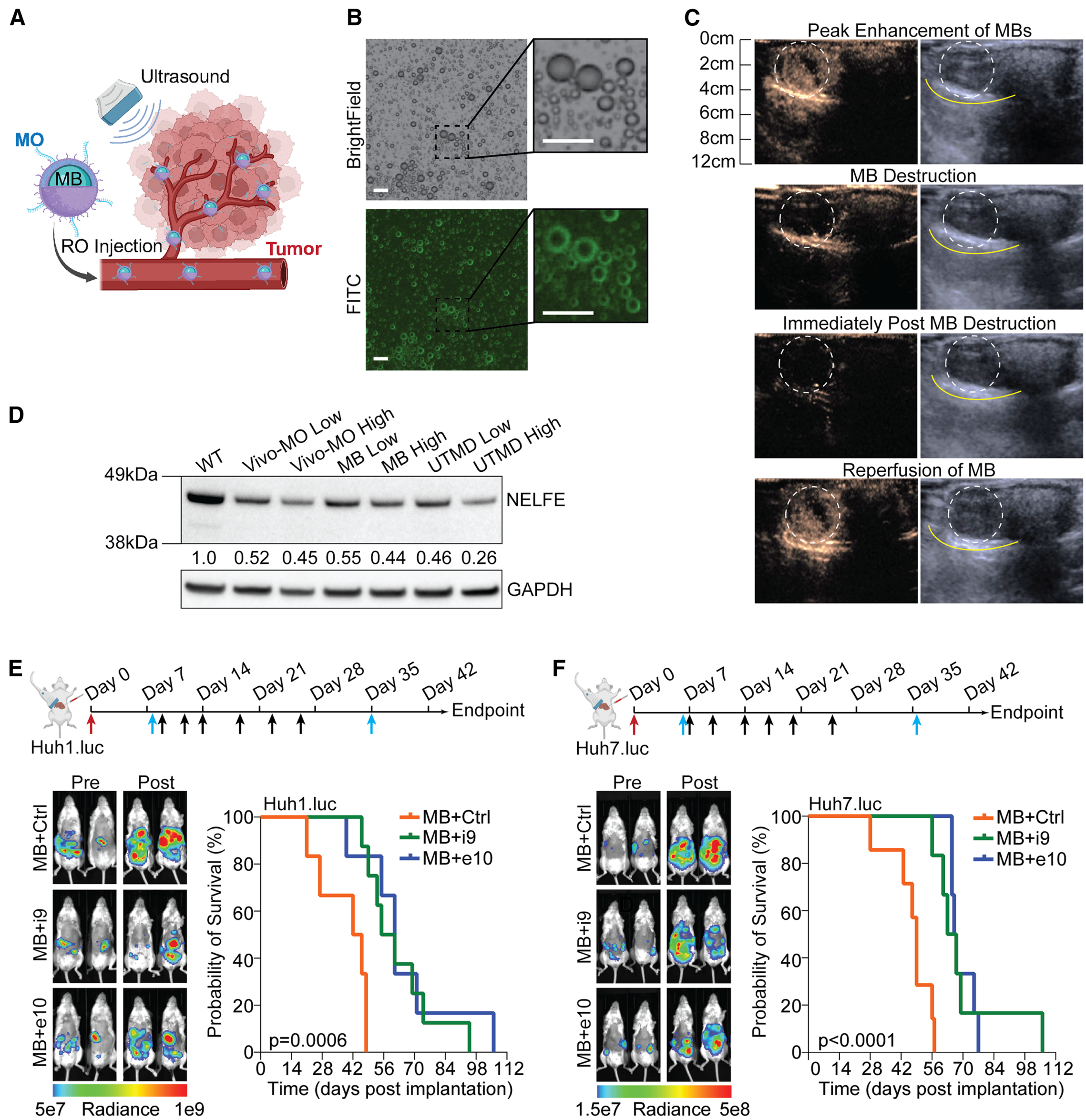

Figure 5. Targeted destruction of NELFE MO-loaded MBs extends survival *in vivo*

(A) Schematic of locally released MOs from UTMD of MO loaded MBs. MO, morpholino; MB, microbubble; RO, retroorbital injection. (B) Microscopy images of Definity MBs loaded with fluoresceinated control vivo-MO. Images taken at 20×. Scale bars, 10 μm. (C) Ultrasound imaging of MB destruction and replenishment sequence. White circle outlines the tumor. Yellow line indicates the liver. (D) Immunoblot of Huh1 spheroids treated with two different concentrations of vivo-MO alone, loaded on MBs without UTMD, or loaded on MBs that subsequently underwent UTMD. NELFE band intensity was normalized to respective GAPDH then to WT. (E and F) Top, schematics of the experimental approach assessing MB + MO effect on Huh1.luc (E) and Huh7.luc (F) orthotopic tumors in NOD/SCID mice. Blue arrows indicate IVIS pre- and post-treatment imaging days. Black arrows indicate treatment days of UTMD of 50 μL MB + 80 μM MO administered through retro-orbital injections. Left, representative bioluminescence images of Huh1.luc (E) and Huh7.luc (F) mice pre-MB+MO treatment and 1-week post- final MB + MO treatment. Scale is in radiance (p/sec/cm/sr). Right, Kaplan-Meier survival curves of Huh1.luc (E) and Huh7.luc (F) vivo-MO treated mice (Huh1.luc experiment: MB + Ctrl *n* = 6, MB + i9 *n* = 8, MB + e10 *n* = 6; Huh7.luc experiment: MB + Ctrl *n* = 7, MB + i9 *n* = 5, MB + e10 *n* = 6). Survival analyses were conducted by a log-rank test.

in 100 μL resuspension buffer (Thermo Fisher Scientific, MPK10025 and N10096) using the Neon Transfection System (Thermo Fisher Scientific, MPK5000) or Neon NxT Electroporation System (Thermo Fisher Scientific, NEON1S) with the protocol 1,275V, 40 pulse width, 1 pulse, and immediately plated in prewarmed media. Cells were used 48 h post electroporation for RNA expression analysis and phenotype assays and 72 h for protein expression analysis.

PCR

RNA was isolated with TRIzol reagent (Invitrogen, 15596018) or using the Direct-zol RNA miniprep kit (Zymogen, 2052). RNA isolated with TRIzol underwent DNase digestion using RQ1 RNase-Free DNase (Promega, M6101). cDNA was synthesized using the high-capacity cDNA reverse transcription kit (Applied Biosystems, 4368814) or the high-capacity RNA-to-cDNA synthesis kit (Applied Biosystems, 4387406). cDNA samples were used for RT-PCR with NELFE SNP F2, NELFE SNP R2, GAPDH F, GAPDH R primers and visualized using the ChemiDoc Imaging System (Bio-Rad) or amplification and detection using CFX Opus 384 Real Time quantification system (Bio-Rad). TaqMan gene expression assays utilized TaqMan Fast Advanced Master Mix (Thermo Fisher Scientific, 4444557) and the following TaqMan probes: 18S (Hs99999901_s1), NELFE exon 10 included (Hs00938402_m1), NELFE d.ex10 (APXG4CC), and NELFE total mRNA (Hs00267952_m1). SyBr Green assays utilized SsoAdvanced Universal SYBR Green Supermix (Bio-Rad, 1725271) and GAPDH F + GAPDH R, NELFE SNP F2 + trunct 1 + 2 seq R, ex8 F + ex9ex10 R, ex8 F + cDNAisoformR2, and SRSF3 F + SRSF3 poison R primer sets. For relative RNA expression, the $2^{-\Delta\Delta Ct}$ method was used with normalization to the endogenous controls 18S for TaqMan assays or GAPDH for SyBr green assays. For d.ex10 isoform analysis, WT or Ctrl Ct was set to the max cycles run, 35. Primer sequences are listed in Table S3.

Western blotting

Cells were lysed in RIPA buffer (Cell Signaling Technology, 9806) supplemented with complete Protease Inhibitor Cocktail (Sigma-Aldrich, 4693116001) and phenylmethylsulfonyl fluoride (Sigma-Aldrich, 11359061001). Protein concentration was determined using the Pierce Rapid Gold BCA Protein Assay Kit (Thermo Fisher, A53225) then samples were prepared with NuPage Sample Reducing Agent (Thermo Fisher Scientific, NP0009) and NuPage LDS Sample Buffer (Thermo Fisher Scientific, NP0007), boiled at 70°C for 10 min, separated on 4%–12% Bis-Tris polyacrylamide gels (Thermo Fisher Scientific, NP0336BOX), and transferred to nitrocellulose membranes using the iBlot dry transfer system (Thermo Fisher Scientific, IB31001). Membranes were blocked in 5% milk for 30 min at room temperature, incubated with primary antibody in 5% milk overnight at 4°C, then incubated with HRP-conjugated anti-rabbit or anti-mouse secondary antibody in 5% milk for 30 min at room temperature. Proteins were visualized with Clarity Western ECL Substrate (Bio-Rad, 1705061) on the ChemiDoc Imaging System (Bio-Rad). The following antibodies were used: anti-NELFE (1:2,000, Abcam, ab170104, RRID:AB_2827280), anti-NELFE (1:2,000, Protein Tech, 10705-1-AP, RRID: AB_513966), anti-NELFE (1:500, Santa Cruz Biotechnology, sc-377052, RRID: AB_2847957), anti-NELFE (1:500, EMD Millipore, ABE48, RRID:AB_10806770), anti-NELFA (1:250, Santa Cruz Biotechnology, sc-365004, RRID:AB_10708864), anti-COBRA1 (1:2,000, Cell Signaling Technology, 14894, RRID:AB_2798637), anti-TH1L (1:2000, Cell Signaling Technology, 12265, RRID:AB_2797862), anti-GAPDH (1:2000, Cell Signaling Technology, 5174, RRID:AB_10622025), anti-U2AF65 (1:5,000, Sigma-Aldrich, U4758, RRID:AB_262122).

Minigene assay

The genomic region from *NELFE* exon 9 through exon 11 was cloned upstream of GFP in the pLenti-c-mGFP backbone (OriGene, PS100071) by In-Fusion Cloning (Takara, 638947). An ATG sequence was added at the beginning of the *NELFE* sequence and the *NELFE* stop codon was removed. A seven amino acid linker sequence was added so that the NELFE and GFP sequences remained in the same reading frame. Mutagenesis PCR introduced the G to An SNP, the product was digested with *Dpn* I (Takara, 1235A) and validated with Sanger-seq. WT or SNP minigene pDNA was transfected into WT HCC cells in a 6-well plate with FuGENE HD transfection reagent (Promega, E2311) and Opti-MEM media, incubated 48 h, then collected in TRIzol reagent. RT-PCR was conducted with Taq 2× master mix (New England Biolabs, M0270L) and NELFE SNP F2 long and NELFE SNP R primers. Bands were visualized using the ChemiDoc Imaging System, purified using Monarch DNA Gel Extraction kit (New England Biolabs, #T1020) and Sanger sequenced (Eurofins Genomics). Primer sequences are listed in Table S3.

Dual luciferase assays

For the minigene dual luciferase reporters, the *NELFE* genomic region was amplified from WT and SNP minigenes (described earlier) and cloned immediately 5′ of *Renilla* luciferase in the linearized psiCHECK-2 backbone (Promega, C8021) using In-Fusion HD cloning. HCC cells were plated in 6-well plates and transfected with 3 μg of dual luciferase minigene reporter using FuGENE HD transfection reagent and opti-MEM (Gibco, 31985070) and incubated for 48 h.

For the 3′ UTR dual luciferase assays, the 3′ UTR sequences were amplified from either Huh1 WT or Huh1 mutant cDNA and cloned immediately 3′ of *Renilla* luciferase's stop codon in the linearized psiCHECK-2 backbone using In-Fusion HD cloning. HCC cells were plated in 6-well plates and transfected with 1 μg of 3′ UTR dual luciferase plasmid DNA using FuGENE HD transfection reagent and opti-MEM (Gibco, 31985070) and incubated for 48 h.

Cells were lysed with 1× passive lysis buffer and dual luciferase assays were conducted following Promega's protocol on a GloMax Explorer. Firefly and *Renilla* expression were normalized to background signal then *Renilla* was normalized to firefly to control for transfection efficiency.

RNA immunoprecipitation qPCR

2.5 × 10 HCC cells were plated in 10 cm plates, transfected with 2.5 μg of minigene DNA, incubated 48 h, crosslinked using formaldehyde (37%) for 10 min room temperature, then incubated 10 min with glycine. Cells were lysed using wash buffer solution (1× PBS, 0.1% SDS, 0.5% deoxycholate, 0.5% NP-40) and incubated on ice for 15 min, then dithiothreitol (Thermo Fisher Scientific, R0861), RiboLock RNase Inhibitor (Thermo Fisher Scientific, EO0382), and RQ1 RNase-Free DNAse (Promega, M6101) were added to the

lysate and incubated 37°C for 5 min at 1,000 rpm then 5 min on ice. Finally, cells were pelleted and the supernatant was used for RNA immunoprecipitation. Dynabeads Protein G beads (Thermo Fisher Scientific, 10004D) were incubated with anti-U2AF65 (2.5 μg, Millipore Sigma, U4758, RRID:AB_262122) or mouse immunoglobulin G (IgG) (2.5 μg, Sigma-Aldrich, 12–371, RRID:AB_145840) antibody on a rotator at room temperature for 30 min to 1 h before lysate was added and incubated on a rotating platform overnight at 4°C. Beads were washed twice with wash buffer, twice with 1× PNK buffer then incubated with 1× PK buffer and 0.2% SDS at 50°C for 1 h. An equal volume of chloroform was added, tubes were centrifuged, and the aqueous phase was kept. Beads were then washed with 100% then 80% ethanol. Pellet was air dried, resuspended in RNAse-free H2O, and used for DNase digestion and cDNA synthesis. cDNA was used with SsoAdvanced SYBR Green Mix and long NELFE SNP F2 long and pLenti-c-seq R primers for qPCR following the SsoAdv_Univ_SYBR_cDNA program on the CFX 384 Opus Real-Time PCR System (Bio-Rad). Primer sequences are listed in Table S3. Relative amount of minigene cDNA pulled down was determined using the $2^{-\Delta\Delta Ct}$ method.

Cycloheximide mRNA surveillance assay

To determine whether the d.ex10 mRNA isoform is subject to translation-dependent mRNA surveillance, cells were treated with the translation inhibitor CHX and transcript abundance was monitored over time. Hep3B cells were transfected with either ctrl or i9 MO and incubated in 6-well plates for 48 h. Culture media was then changed to PS free containing CHX at a final concentration of 0.1 mg/mL. Cells were harvested in TRIzol at 0, 1, 2, 4, and 6 h of CHX-treatment. Transcript levels were assessed by quantitative reverse-transcription PCR (RT-qPCR) using SsoAdvanced SYBR Green Mix with isoform specific primers: ex8 F + ex9ex10 R for exon 10 included transcripts or ex8 F + cDNAisoformR2 (spans exon9-exon11 junction) for exon 10 excluded transcripts. The SRSF3 poison exon was used as a positive experimental control as it is known to undergo NMD. Primer sequences are listed in Table S3.

mRNA stability analysis

Huh1 WT and Huh1 d.ex10 mutant cells were seeded in 6 cm plates and incubated for 48 h. Cells were serum deprived in DMEM supplemented with 0.5% cFBS and 1% LG for 12 h before media was replaced with DMEM supplemented with 1% LG, 10% cFBS, and 60 μM DRB (Sigma-Aldrich, D1916). Following MO transfection into Huh1 cells, cells were seeded in 6-well plates and incubated 48 h before media was replaced with DMEM supplemented with 1% LG, 10% cFBS and 5 μg/mL act. D (Thermo Fisher Scientific, 11805017). Cells were collected in TRIzol at 0, 2, 4, 6, or 8 h of transcription inhibition treatment. RT-qPCR analysis was performed using TaqMan gene expression probes for exon 10 included (Thermo Fisher Scientific, Hs00938402_m1) and exon 10 excluded (Thermo Fisher Scientific, APXG4CC) isoforms and relative mRNA analysis was performed using the $2^{-\Delta\Delta Ct}$ method with 18S (Thermo Fisher Scientific, Hs99999901_s1) as the endogenous control. mRNA levels were calibrated to the 0 h time point.

siNELFE assay

To determine what bands on NELFE immunoblots were *NELFE* derived, siRNAs targeting different regions of *NELFE* were transfected into Huh1 WT and Huh1 d.ex10 mut cells. 100 K WT cells or 130 K mut cells were plated in 6-well plates and incubated 24 h. Cells were then forward transfected with RNAiMAX and either 10 nM of FlexiTube siRNA Hs_RDBP_4 (targets exon 4, GeneGlobe ID: SI00700868, QIAGEN) or Hs_RDBP_3 (targets exon 10, GeneGlobe ID: SI00700861, QIAGEN) and incubated 72 h before cells were lysed for immunoblotting. NELFE protein lacking exon 10 (d.ex10) would be knocked down with the siRNA targeting exon 4 but not the siRNA targeting exon 10 sequences.

Mass spectrometry

Five million WT or NELFE d.ex10 mutant (mut) Huh1 cells were seeded in 15 cm dishes. The next day, cells were washed three times with ice-cold 1× PBS, scraped, and lysed in 1× RIPA buffer supplemented with phenylmethylsulfonyl fluoride. Lysates were incubated on ice for 10 min and subsequently sonicated (1 s ON/1 s OFF, 40% amplitude) on ice. Following sonication, lysates were incubated on ice for an additional 10 min and clarified by centrifugation at 12,000 rpm for 20 min at 4°C. Approximately 7 mg of total protein was used for each immunoprecipitation. For antibody binding, 50 μL of Dynabeads Protein G (Thermo Fisher Scientific, Cat. #10004D) were incubated with 5 μg of anti-NELFE antibody (Proteintech, Cat. #10705-1-AP) or 5 μg of normal rabbit IgG (Millipore, Cat. #12–370) for 2 h at 4°C with rotation. Antibodies were then crosslinked to the beads using 5 mM BS3 (Thermo Fisher Scientific, Cat. #21580) freshly prepared in 1× PBS (pH 8.0) for 10 min at room temperature with rotation. The crosslinking reaction was quenched by adding glycine to a final concentration of 125 mM and incubating for 10 min at room temperature with rotation. Equal amounts of clarified lysate were added to the beads with the coupled antibody and incubated overnight at 4°C with rotation. The following day, beads were washed five times with 1× TBS. Bound proteins were eluted by incubation in 1× PNK buffer containing 1× LDS Sample Buffer (Thermo Fisher Scientific) and 1× Sample Reducing Agent (Thermo Fisher Scientific) for 10 min at 70°C. Eluted proteins were separated on 12% Bis-Tris 12-well gels using 1× MOPS SDS Running Buffer (Thermo Fisher Scientific, Cat. #NP0001) at 160 V for 4 h. After the electrophoresis, gels were stored in nuclease-free water at 4°C until submission to the Wistar Proteomics Facility for mass spectrometry analysis. Three biological replicates were utilized.

NELFE rescue assay

Hep3B cells were seeded at a density of 1 × 10 cells per well in 6-well plates and transduced with lentiviral particles carrying either pLKO.1 shNELFE (target sequence: 5′-CTGGATTCCTTGTGCCT-CATA-3′) or the corresponding pLKO.1 scramble control (target sequence: 5′-CGGCAAGAAGAGATTAGCAAA-3′). Forty-eight hours after transduction, cells were selected with puromycin for 10 days. NELFE knockdown was confirmed by immunoblotting before the rescue experiments.

Following knockdown validation, 1 × 10 cells were replated in 6-well plates and transduced with lentiviral vectors expressing full-length NELFE (pLenti-NELFE FL; OriGene, Cat. No. RC208658L2), NELFE lacking exon 10 (pLenti-NELFE Δexon10; generated by In-Fusion cloning using the In-Fusion HD Cloning Kit, Takara, Cat. No. 638948), or the corresponding empty vector (pLenti-EV). Polybrene (MilliporeSigma, Cat. No. TR-1003) was added at a final concentration of 8 μg/mL at the time of transduction to enhance viral infection. The culture medium was replaced 24 h later. 48 h after rescue transduction, cells were collected, and 2,500 cells per well were seeded into E-Plates (Agilent Technologies, Cat. No. 300601150). Cell proliferation was monitored continuously for 96 h using the xCELLigence Real-Time Cell Analysis system (Agilent Technologies), and cell index values were recorded throughout the experiment. Experiments were performed using at least three independent biological replicates, with each condition measured in technical triplicate.

Cell proliferation assay

2.0 × 10 Hep3B, 2.0 × 10 Huh1, 1.0 × 10 HLE, 3.0 × 10 Huh7, and 4.0 × 10 MHCC97H cells were plated in triplicate or quadruplet in E-Plate View 16 plates (Agilent Technologies, 300601140) and cell proliferation measurements were taken every 15 min by the xCELLigence Real-Time Cell Analysis dual purpose machine (Agilent Technologies).

Cell colony formation assay

2.0 × 10 Hep3B, 2.0 × 10 Huh1, 1.0 × 10 HLE, 3.0 × 10 Huh7, and 2.0 × 10 MHCC97H cells were plated in triplicate in 6-well plates and incubated at 37°C with 5% CO_2 for 10–14 days. Cells were washed with 1× PBS, fixed in 10% formalin for 15 min, washed with 1× PBS, then stained with 0.05% crystal violet dye for 2 h. Colonies were imaged using the ChemiDoc Imaging System (Bio-Rad) and manually counted.

Spheroid formation assay

6×10^{4} Huh7 and 5 × 10 Huh1 ASO-treated cells were mixed with 10% AlgiMatrix firming buffer (Thermo Fisher Scientific, A1091501), plated on AlgiMatrix 6-well, 3D culturing system (Thermo Fisher Scientific, A1098202), and incubated 14 days. To collect spheroids, the AlgiMatrix was constantly agitated with AlgiMatrix dissolving buffer (Thermo Fisher Scientific, A1134001), centrifuged, and spheroids were plated in triplicate on coated (0.1 mg/mL fibronectin, 0.3 mg/mL bovine collagen type 1, 0.1 mg/mL bovine serum albumin in culture medium) 6-well plates for brightfield imaging on an EVOS FL Imager (Life Technologies) and crystal violet staining.

To evaluate the efficacy of UTMD delivery of vivo-MO compared to controls, 150,000 Huh1 cells were mixed with 10% AlgiMatrix firming buffer, plated on AlgiMatrix 6-well, 3D culturing system and incubated for 7 days. At the end of day 6, matrixes were moved to a new 6-well dish and serum free media + 1% LG. On day 7, media was removed and 1 mL of serum free media was added. 40 or 80 μM of vivo-ASO was added to Definity MB vials, which were activated via vigorous shaking for 45 s 50 μL of activated MB were added to the media and either subjected to UTMD using color Doppler imaging with a curved array probe (imaging parameters: transmit frequency 1.5 MHz, MI = 1.4, pulse repetition frequency at 237 HZ) for 5 min to stimulate the UTMD condition or remained untouched for the vivo-MO+MB condition. The vivo-MO alone condition received the same final concentration of vivo-MO in media as the MB conditions (2 or 4 μM). An additional 1 mL of serum free media was added to wells at the end of day 7. Media was changed to serum containing + 1% LG media the following day. Spheroids were collected for RNA and protein analysis 72 h post treatment.

MB-MO microscopy

Fluoresceinated standard control MOs (50 μL, 0.5 mM) were conjugated to Definity MBs during MB activation. For visualization, 10–20 μL of MO-loaded MBs were deposited onto a slide, cover slipped and immediately imaged at 20× magnification using a Nikon Ni-U microscope. Fluorescence was analyzed using the Nikon NIS-Elements analysis software.

Immunohistochemistry

Paraffin Blocks were sectioned at 5 μm and stained with hematoxylin and eosin or anti-NELFE (1:500, Protein Tech, 10705-1-AP, RRID: AB_513966). Immunohistochemistry of human samples was cross validated by two pathologists.

Statistical analysis

Statistical analysis and data visualization was conducted using GraphPad Prism (v.10, RRID:SCR_002798). Unpaired Student's *t* tests or Welch's test were used to compare two groups. Analysis was performed with at least 3 technical replicates, and experiments were conducted with at least three biological replicates. For more than two groups, one-way ANOVAs were used with either Tukey's or Holm-Šídák's multiple comparisons test. For comparing measurements over time between two or more groups, two-way ANOVAs were used with Dunnett's multiple comparisons tests. Log-rank tests were utilized for survival analyses. p value <0.05 were considered statistically significant.

DATA AND CODE AVAILABILITY

Data from this study is available upon request from the corresponding author.

ACKNOWLEDGMENTS

Research reported in this publication was supported by the National Cancer Institute at the National Institutes of Health under award number R01CA284017, the American Liver Foundation, and the WW Smith Trust Foundation. The content is solely the responsibility of the authors and does not necessarily represent the official views of the National Institutes of Health. We acknowledge the use of the Translational Pathology Shared Resource of the Sidney Kimmel Comprehensive Cancer Center, Thomas Jefferson University, to conduct this research (supported by NCI grant 5P30CA056036). We would also like to acknowledge The Wistar Institute's Proteomics & Metabolomics Shared resource for providing technical support. We would like to acknowledge the Sidney Kimmel Comprehensive Cancer Center's Working Liver Group for their support and collaboration to conduct this research. Funding support for The Wistar Institute core facilities was provided by Cancer Center support grant P30CA010815. M.F. and X.W.W. were supported by the Intramural Research Program of the Center for Cancer Research, National Cancer Institute, National Institutes of Health. The contributions of the NIH authors were made as part of their official duties as NIH federal employees, are

in compliance with agency policy requirements, and are considered Works of the United States Government. However, views and interpretations presented in this paper are those of the author(s) and do not necessarily reflect the views of the NIH or the US Department of Health and Human Services.

AUTHOR CONTRIBUTIONS

L.M.R. generated the NELFE d.ex10 cell line and conducted all relevant assays, cloned, and performed the *NELFE* 3′ UTR dual luciferase assay, and carried out all animal experiments. T.N. assisted in generating the NELFE d.ex10 cell line. L.M.R., R.L., and G.M.P. performed *in vitro* ASO experiments. P.C. performed ENCODE CLIP-seq analysis. R.L. and H.D. performed patient tissue qPCR. K.Z. and A.E.B.-W. generated and performed the pLenti-minigene reporter and the dual luciferase minigene reporter assays. H.D. performed the RIP-qPCR. L.M.R. and K.M.H. conducted siNELFE and ASO time course assays. A.L. prepared samples for mass spectrometry. C.E.W. and J.-B.L. performed ultrasound guided cell injections and UTMD. A.L. and J.R.E. conducted MB injections. B.N.R. assisted with animal experiments and provided conceptual support. C.E. and M.M.G. cross validated the immunohistochemistry of patient tissue. K.Y.H. and R.L. collected patient samples, and L.M.R. and K.Y.H. maintained clinical data. R.L., A.S.B., A.P.S., W.J., and D.L. provided HCC patient samples and interpretation and analyses of clinical data. M.F. and X.W.W. initially identified the SNP. N.L.F., S.P.M., M.M.G., and J.R.E. provided collaborative and conceptual support. L.M.R. drafted the manuscript. H.D. conceived the idea, supervised the project, and acquired funding. All authors read and edited the manuscript.

DECLARATION OF INTERESTS

The authors declare a patent application pending (#64/102,255) for the MO + ASO technology.

SUPPLEMENTAL INFORMATION

Supplemental information can be found online at https://doi.org/10.1016/j.omtn.2026.103124.

REFERENCES

1. Rumgay, H., Arnold, M., Ferlay, J., Lesi, O., Cabasag, C.J., Vignat, J., Laversanne, M., McGlynn, K.A., and Soerjomataram, I. (2022). Global burden of primary liver cancer in 2020 and predictions to 2040. J. Hepatol. *77*, 1598–1606. https://doi.org/10.1016/j.jhep.2022.08.021.
2. Bray, F., Laversanne, M., Sung, H., Ferlay, J., Siegel, R.L., Soerjomataram, I., and Jemal, A. (2024). Global cancer statistics 2022: GLOBOCAN estimates of incidence and mortality worldwide for 36 cancers in 185 countries. CA Cancer J. Clin. *74*, 229–263. https://doi.org/10.3322/caac.21834.
3. Rumgay, H., Ferlay, J., De Martel, C., Georges, D., Ibrahim, A.S., Zheng, R., Wei, W., Lemmens, V.E.P.P., and Soerjomataram, I. (2022). Global, regional and national burden of primary liver cancer by subtype. Eur. J. Cancer *161*, 108–118. https://doi.org/10.1016/j.ejca.2021.11.023.
4. Siegel, R.L., Giaquinto, A.N., and Jemal, A. (2024). Cancer statistics, 2024. CA Cancer J. Clin. *74*, 12–49. https://doi.org/10.3322/caac.21820.
5. Safri, F., Nguyen, R., Zerehpooshnesfchi, S., George, J., and Qiao, L. (2024). Heterogeneity of hepatocellular carcinoma: from mechanisms to clinical implications. Cancer Gene Ther. *31*, 1105–1112. https://doi.org/10.1038/s41417-024-00764-w.
6. Amin, N., Anwar, J., Sulaiman, A., Naumova, N.N., and Anwar, N. (2025). Hepatocellular Carcinoma: A Comprehensive Review. Diseases *13*, 207. https://doi.org/10.3390/diseases13070207.
7. Tümen, D., Heumann, P., Gülow, K., Demirci, C.-N., Cosma, L.-S., Müller, M., and Kandulski, A. (2022). Pathogenesis and Current Treatment Strategies of Hepatocellular Carcinoma. Biomedicines *10*, 3202. https://doi.org/10.3390/biomedicines10123202.
8. Dang, H., Takai, A., Forgues, M., Pomyen, Y., Mou, H., Xue, W., Ray, D., Ha, K.C.H., Morris, Q.D., and Hughes, T.R. (2017). Oncogenic Activation of the RNA Binding Protein NELFE and MYC Signaling in Hepatocellular Carcinoma. Cancer Cell *32*, 101–114.e8. https://doi.org/10.1016/j.ccell.2017.06.002.
9. Zhang, B., Babu, K.R., Lim, C.Y., Kwok, Z.H., Li, J., Zhou, S., Yang, H., and Tay, Y. (2020). A comprehensive expression landscape of RNA-binding proteins (RBPs) across 16 human cancer types. RNA Biol. *17*, 211–226. https://doi.org/10.1080/15476286.2019.1673657.
10. Narita, T., Yamaguchi, Y., Yano, K., Sugimoto, S., Chanarat, S., Wada, T., Kim, D.k., Hasegawa, J., Omori, M., Inukai, N., et al. (2003). Human Transcription Elongation Factor NELF: Identification of Novel Subunits and Reconstitution of the Functionally Active Complex. Mol. Cell Biol. *23*, 1863–1873. https://doi.org/10.1128/MCB.23.6.1863-1873.2003.
11. Core, L., and Adelman, K. (2019). Promoter-proximal pausing of RNA polymerase II: a nexus of gene regulation. Genes Dev. *33*, 960–982. https://doi.org/10.1101/gad.325142.119.
12. Schulze, W.M., and Cusack, S. (2017). Structural basis for mutually exclusive co-transcriptional nuclear cap-binding complexes with either NELF-E or ARS2. Nat. Commun. *8*, 1302. https://doi.org/10.1038/s41467-017-01402-w.
13. Aoi, Y., Smith, E.R., Shah, A.P., Rendleman, E.J., Marshall, S.A., Woodfin, A.R., Chen, F.X., Shiekhattar, R., and Shilatifard, A. (2020). NELF Regulates a Promoter-Proximal Step Distinct from RNA Pol II Pause-Release. Mol. Cell *78*, 261–274.e5. https://doi.org/10.1016/j.molcel.2020.02.014.
14. Chen, C., Zheng, Q., Pan, S., Chen, W., Huang, J., Cao, Y., Tu, Y., Li, Z., Yu, C., and Jie, Z. (2021). The RNA-Binding Protein NELFE Promotes Gastric Cancer Growth and Metastasis Through E2F2. Front. Oncol. *11*, 677111. https://doi.org/10.3389/fonc.2021.677111.
15. Han, L., Zan, Y., Huang, C., and Zhang, S. (2019). NELFE promoted pancreatic cancer metastasis and the epithelial-to-mesenchymal transition by decreasing the stabilization of NDRG2 mRNA. Int. J. Oncol. *55*, 1313–1323. https://doi.org/10.3892/ijo.2019.4890.
16. Yu, S., Li, L., Cai, H., He, B., Gao, Y., and Li, Y. (2021). Overexpression of NELFE contributes to gastric cancer progression via Wnt/β-catenin signaling-mediated activation of CSNK2B expression. J. Exp. Clin. Cancer Res. *40*, 54. https://doi.org/10.1186/s13046-021-01848-3.
17. Zhang, J., Hu, Z., Chung, H.H., Tian, Y., Lau, K.W., Ser, Z., Lim, Y.T., Sobota, R.M., Leong, H.F., Chen, B.J., et al. (2023). Dependency of NELF-E-SLUG-KAT2B epigenetic axis in breast cancer carcinogenesis. Nat. Commun. *14*, 2439. https://doi.org/10.1038/s41467-023-38132-1.
18. Xue, Y., Liu, L., Yu, L., Li, Z., Huang, X., Chen, S., Jing, Y., Zhang, X., Ding, L., Song, Y., et al. (2025). Highly Expressed NELFE in Tumor Cells at the Invasive Tumor Front Was a Prognostic Biomarker for Oral Squamous Cell Carcinoma. J. Oral Pathol. Med. *54*, 757–769. https://doi.org/10.1111/jop.70025.
19. Schulze, K., Imbeaud, S., Letouzé, E., Alexandrov, L.B., Calderaro, J., Rebouissou, S., Couchy, G., Meiller, C., Shinde, J., Soysouvanh, F., et al. (2015). Exome sequencing of hepatocellular carcinomas identifies new mutational signatures and potential therapeutic targets. Nat. Genet. *47*, 505–511. https://doi.org/10.1038/ng.3252.
20. Singal, A.G., Llovet, J.M., Yarchoan, M., Mehta, N., Heimbach, J.K., Dawson, L.A., Jou, J.H., Kulik, L.M., Agopian, V.G., Marrero, J.A., et al. (2023). AASLD Practice Guidance on prevention, diagnosis, and treatment of hepatocellular carcinoma. Hepatology *78*, 1922–1965. https://doi.org/10.1097/HEP.0000000000000466.
21. Cucarull, B., Tutusaus, A., Rider, P., Hernáez-Alsina, T., Cuño, C., García de Frutos, P., Colell, A., Marí, M., and Morales, A. (2022). Hepatocellular Carcinoma: Molecular Pathogenesis and Therapeutic Advances. Cancers *14*, 621. https://doi.org/10.3390/cancers14030621.
22. Rimassa, L., Finn, R.S., and Sangro, B. (2023). Combination immunotherapy for hepatocellular carcinoma. J. Hepatol. *79*, 506–515. https://doi.org/10.1016/j.jhep.2023.03.003.
23. Pinter, M., Trauner, M., Peck-Radosavljevic, M., and Sieghart, W. (2016). Cancer and liver cirrhosis: implications on prognosis and management. ESMO Open *1*, e000042. https://doi.org/10.1136/esmoopen-2016-000042.
24. Kooiman, K., Roovers, S., Langeveld, S.A.G., Kleven, R.T., Dewitte, H., O'Reilly, M.A., Escoffre, J.-M., Bouakaz, A., Verweij, M.D., Hynynen, K., et al. (2020). Ultrasound-Responsive Cavitation Nuclei for Therapy and Drug Delivery. Ultrasound Med. Biol. *46*, 1296–1325. https://doi.org/10.1016/j.ultrasmedbio.2020.01.002.
25. Tu, J., and Yu, A.C.H. (2022). Ultrasound-Mediated Drug Delivery: Sonoporation Mechanisms, Biophysics, and Critical Factors. BME Front. *2022*, 9807347. https://doi.org/10.34133/2022/9807347.
26. Yang, B.-J., Zhou, H.-F., Liu, Y., Yang, W., Li, J., and Zhou, W.-Z. (2025). The current progress and futural prospects of ultrasound-targeted microbubble destruction

for liver diseases: a narrative review. Transl. Gastroenterol. Hepatol. *10*, 14. https://doi.org/10.21037/tgh-24-53.

27. Lu, K., Fan, Q., and Zou, X. (2022). Antisense oligonucleotide is a promising intervention for liver diseases. Front. Pharmacol. *13*, 1061842. https://doi.org/10.3389/fphar.2022.1061842.

28. Havens, M.A., and Hastings, M.L. (2016). Splice-switching antisense oligonucleotides as therapeutic drugs. Nucleic Acids Res. *44*, 6549–6563. https://doi.org/10.1093/nar/gkw533.

29. Li, Y.-F., and Morcos, P.A. (2008). Design and Synthesis of Dendritic Molecular Transporter that Achieves Efficient in Vivo Delivery of Morpholino Antisense Oligo. Bioconjug. Chem. *19*, 1464–1470. https://doi.org/10.1021/bc8001437.

30. Moulton, J. (2016). Guide for Morpholino Users: Toward Therapeutics. J. Drug Discov. Dev. Deliv. *3*, 1023.

31. Muppirala, U.K., Honavar, V.G., and Dobbs, D. (2011). Predicting RNA-Protein Interactions Using Only Sequence Information. BMC Bioinf. *12*, 489. https://doi.org/10.1186/1471-2105-12-489.

32. Muppirala, U.K., Lewis, B.A., and Dobbs, D. (2013). Computational Tools for Investigating RNA-Protein Interaction Partners. J Comput Sci Syst Biol *06*. https://doi.org/10.4172/jcsb.1000114.

33. Zhang, Y., Qian, J., Gu, C., and Yang, Y. (2021). Alternative splicing and cancer: a systematic review. Sig Transduct Target Ther *6*, 78. https://doi.org/10.1038/s41392-021-00486-7.

34. Wahl, M.C., Will, C.L., and Lührmann, R. (2009). The Spliceosome: Design Principles of a Dynamic RNP Machine. Cell *136*, 701–718. https://doi.org/10.1016/j.cell.2009.02.009.

35. Tao, Y., Zhang, Q., Wang, H., Yang, X., and Mu, H. (2024). Alternative splicing and related RNA binding proteins in human health and disease. Sig Transduct Target Ther *9*, 26–33. https://doi.org/10.1038/s41392-024-01734-2.

36. Hong, D., and Jeong, S. (2023). 3'UTR Diversity: Expanding Repertoire of RNA Alterations in Human mRNAs. Mol. Cells *46*, 48–56. https://doi.org/10.14348/molcells.2023.0003.

37. Sun, J., Watkins, G., Blair, A.L., Moskaluk, C., Ghosh, S., Jiang, W.G., and Li, R. (2008). Deregulation of cofactor of BRCA1 expression in breast cancer cells. J. Cell. Biochem. *103*, 1798–1807. https://doi.org/10.1002/jcb.21568.

38. Sun, J., and Li, R. (2010). Human Negative Elongation Factor Activates Transcription and Regulates Alternative Transcription Initiation. J. Biol. Chem. *285*, 6443–6452. https://doi.org/10.1074/jbc.M109.084285.

39. Rao, J.N., Schweimer, K., Wenzel, S., Wöhrl, B.M., and Rösch, P. (2008). NELF-E RRM Undergoes Major Structural Changes in Flexible Protein Regions on Target RNA Binding. Biochemistry *47*, 3756–3761. https://doi.org/10.1021/bi702429m.

40. Iida, M., Iizuka, N., Tsunedomi, R., Tsutsui, M., Yoshida, S., Maeda, Y., Tokuhisa, Y., Sakamoto, K., Yoshimura, K., Tamesa, T., et al. (2012). Overexpression of the RD RNA binding protein in hepatitis C virus-related hepatocellular carcinoma. Oncol. Rep. *28*, 728–734. https://doi.org/10.3892/or.2012.1821.

41. Morcos, P.A., Li, Y., and Jiang, S. (2008). Vivo-Morpholinos: A non-peptide transporter delivers Morpholinos into a wide array of mouse tissues. Biotechniques *45*, 613–623. https://doi.org/10.2144/000113005.

42. Rawat, P., Boehning, M., Hummel, B., Aprile-Garcia, F., Pandit, A.S., Eisenhardt, N., Khavaran, A., Niskanen, E., Vos, S.M., Palvimo, J.J., et al. (2021). Stress-induced nuclear condensation of NELF drives transcriptional downregulation. Mol. Cell *81*, 1013–1026.e11. https://doi.org/10.1016/j.molcel.2021.01.016.

43. Yamaguchi, Y., Takagi, T., Wada, T., Yano, K., Furuya, A., Sugimoto, S., Hasegawa, J., and Handa, H. (1999). NELF, a Multisubunit Complex Containing RD, Cooperates with DSIF to Repress RNA Polymerase II Elongation. Cell *97*, 41–51. https://doi.org/10.1016/S0092-8674(00)80713-8.

44. Sharma, D., and Czarnota, G.J. (2025). Using ultrasound and microbubble to enhance the effects of conventional cancer therapies in clinical settings. Cancer Metastasis Rev. *44*, 39. https://doi.org/10.1007/s10555-025-10255-5.

45. Wessner, C.E., Forsberg, F., Lyshchik, A., O'Kane, P., Bradigan, K., Liu, J.-B., Delaney, L.J., Anton, K., Topper, S.R., Civan, J., et al. (2025). Microbubble-based Radiosensitization of Hepatocellular Carcinoma: Evaluation of Safety and Efficacy in a Phase II Randomized Trial. Radiology *316*, e250083. https://doi.org/10.1148/radiol.250083.

46. Keller, S.B., Suo, D., Wang, Y.-N., Kenerson, H., Yeung, R.S., and Averkiou, M.A. (2020). Image-Guided Treatment of Primary Liver Cancer in Mice Leads to Vascular Disruption and Increased Drug Penetration. Front. Pharmacol. *11*, 584344. https://doi.org/10.3389/fphar.2020.584344.

47. Cochran, M.C., Eisenbrey, J.R., Soulen, M.C., Schultz, S.M., Ouma, R.O., White, S.B., Furth, E.E., and Wheatley, M.A. (2011). Disposition of Ultrasound Sensitive Polymeric Drug Carrier in a Rat Hepatocellular Carcinoma Model. Acad. Radiol. *18*, 1341–1348. https://doi.org/10.1016/j.acra.2011.06.013.

48. Jing, H., Cheng, W., Zhang, J.-W., Han, X., Shao, H., and Sun, Y.-X. (2015). Galactosylated poly-L-lysine targeted microbubbles for ultrasound mediated antisense c-myc gene transfection in hepatocellular carcinoma cells. aoms *11*, 292–300. https://doi.org/10.5114/aoms.2015.50963.

49. Morcos, P.A. (2007). Achieving targeted and quantifiable alteration of mRNA splicing with Morpholino oligos. Biochem. Biophys. Res. Commun. *358*, 521–527. https://doi.org/10.1016/j.bbrc.2007.04.172.

50. Sang, A., Zhuo, S., Bochanis, A., Manautou, J.E., Bahal, R., Zhong, X.B., and Rasmussen, T.P. (2024). Mechanisms of Action of the US Food and Drug Administration-Approved Antisense Oligonucleotide Drugs. BioDrugs *38*, 511–526. https://doi.org/10.1007/s40259-024-00665-2.

51. Hui, R.W.-H., Mak, L.-Y., Seto, W.-K., and Yuen, M.-F. (2022). RNA interference as a novel treatment strategy for chronic hepatitis B infection. CMH *28*, 408–424. https://doi.org/10.3350/cmh.2022.0012.

52. Caddeo, A., and Romeo, S. (2025). Precision medicine and nucleotide-based therapeutics to treat steatotic liver disease. CMH *31*, S76–S93. https://doi.org/10.3350/cmh.2024.0438.

53. Allen, R., and Yokota, T. (2024). Endosomal Escape and Nuclear Localization: Critical Barriers for Therapeutic Nucleic Acids. Molecules *29*, 5997. https://doi.org/10.3390/molecules29245997.

54. Ou, J., Guan, X., Wang, J., Wang, T., Zhang, B., Li, R., Xu, H., Hu, X., and Guo, X.-K. (2022). Epithelial NELF guards intestinal barrier function to ameliorate colitis by maintaining junctional integrity. Mucosal Immunol. *15*, 279–288. https://doi.org/10.1038/s41385-021-00465-9.

55. Robinson, D.C.L., Ritso, M., Nelson, G.M., Mokhtari, Z., Nakka, K., Bandukwala, H., Goldman, S.R., Park, P.J., Mounier, R., Chazaud, B., et al. (2021). Negative elongation factor regulates muscle progenitor expansion for efficient myofiber repair and stem cell pool repopulation. Dev. Cell *56*, 1014–1029.e7. https://doi.org/10.1016/j.devcel.2021.02.025.

56. Geary, R.S., Norris, D., Yu, R., and Bennett, C.F. (2015). Pharmacokinetics, biodistribution and cell uptake of antisense oligonucleotides. Adv. Drug Deliv. Rev. *87*, 46–51. https://doi.org/10.1016/j.addr.2015.01.008.

57. Cerroni, B., Cicconi, R., Oddo, L., Scimeca, M., Bonfiglio, R., Bernardini, R., Palmieri, G., Domenici, F., Bonanno, E., Mattei, M., et al. (2018). In vivo biological fate of poly(vinylalcohol) microbubbles in mice. Heliyon *4*, e00770. https://doi.org/10.1016/j.heliyon.2018.e00770.

58. Roessler, S., Jia, H.-L., Budhu, A., Forgues, M., Ye, Q.-H., Lee, J.-S., Thorgeirsson, S.S., Sun, Z., Tang, Z.-Y., Qin, L.-X., et al. (2010). A Unique Metastasis Gene Signature Enables Prediction of Tumor Relapse in Early-Stage Hepatocellular Carcinoma Patients. Cancer Res. *70*, 10202–10212. https://doi.org/10.1158/0008-5472.CAN-10-2607.

59. Diehl, K.-H., Hull, R., Morton, D., Pfister, R., Rabemampianina, Y., Smith, D., Vidal, J.-M., and van de Vorstenbosch, C.; European Federation of Pharmaceutical Industries Association and European Centre for the Validation of Alternative Methods (2001). A good practice guide to the administration of substances and removal of blood, including routes and volumes. J. Appl. Toxicol. *21*, 15–23. https://doi.org/10.1002/jat.727.

60. Diagnostic Ultrasound Enhancing Agent | DEFINITY® (Perflutren Lipid Microsphere) https://www.definityimaging.com/.

61. Jumper, J., Evans, R., Pritzel, A., Green, T., Figurnov, M., Ronneberger, O., Tunyasuvunakool, K., Bates, R., Žídek, A., Potapenko, A., et al. (2021). Highly accurate protein structure prediction with AlphaFold. Nature *596*, 583–589. https://doi.org/10.1038/s41586-021-03819-2.

OMTN, Volume *37*

Supplemental information

Morpholino-mediated splice switching of NELFE suppresses hepatocellular carcinoma cell phenotypes *in vitro* and *in vivo*

Laura M. Reynolds, Ryan Lamm, Kai Zhang, Anna E. Barry-Wolbers, Alvaro Lucci, Brittany N. Ruiz, Corinne E. Wessner, Trish Nguyen, Kayla M. Hammond, Christoph Eckert, Ji-Bin Liu, Pongsakorn Choochuen, Kai Ying Hong, Wei Jiang, Marshonna Forgues, Gaia M. Palazzo, Daniel Lin, Adam S. Bodzin, Ashesh P. Shah, Xin Wei Wang, Sean P. Martin, Nicolas L. Fawzi, Matthias M. Gaida, John R. Eisenbrey, and Hien Dang

Supplemental Materials

Characteristic	WT (n=73)	SNP (n=8)
Age		
Mean (SD)	52.6 (13.3)	56.0 (8.6)
BMI		
Mean (SD)	23.0 (3.4)	24.9 (2.2)
Pre-op ALT (IU/L)		
Mean (SD)	77.3 (136.5)	41.0 (12.2)
Pre-op AFP (ng/mL)		
Mean (SD)	12,268.3 (22,606.1)	160.0 (423.1)
Main Tumor Size (cm)		
Mean (SD)	7.11 (3.44)	4.23 (1.80)
Gender		
Male	65 (89%)	6 (75%)
Female	8 (11%)	2 (25%)
HBV		
Yes	69 (94.5%)	7 (87.5%)
No	4 (5.5%)	1 (12.5%)
HCV		
Yes	2 (2.7%)	1 (12.5%)
No	71 (97.3%)	7 (87.5%)
Cirrhosis		
Yes	55 (75.3%)	7 (87.5%)
No	18 (24.7%)	1 (12.5%)
Surgical Procedure		
Transplant	5 (6.8%)	0 (0%)
Resection	68 (93.2%)	8 (100%)
Previous Treatment		
Yes	8 (11%)	0 (0%)
No	65 (89%)	8 (100%)
Satellite Nodule		
Yes	14 (19.2%)	1 (12.5%)
No	59 (80.8%)	7 (87.5%)
Multinodular		
Yes	24 (32.9%)	1 (12.5%)
No	49 (67.1%)	7 (87.5%)
Invasion of Lymph Nodes		
Yes	0 (0%)	0 (0%)
No	73 (100%)	8 (100%)
TNM Stage		
I	40 (54.8%)	8 (100%)
II	10 (13.7%)	0 (0%)
III	23 (31.5%)	0 (0%)
IV	0 (0%)	0 (0%)

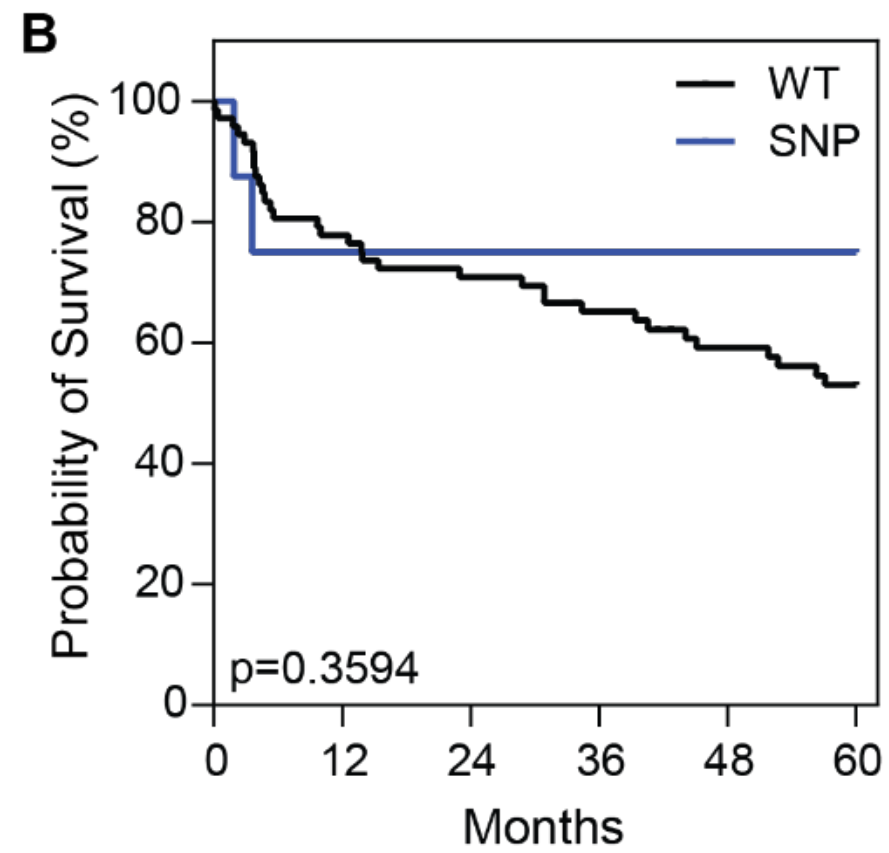

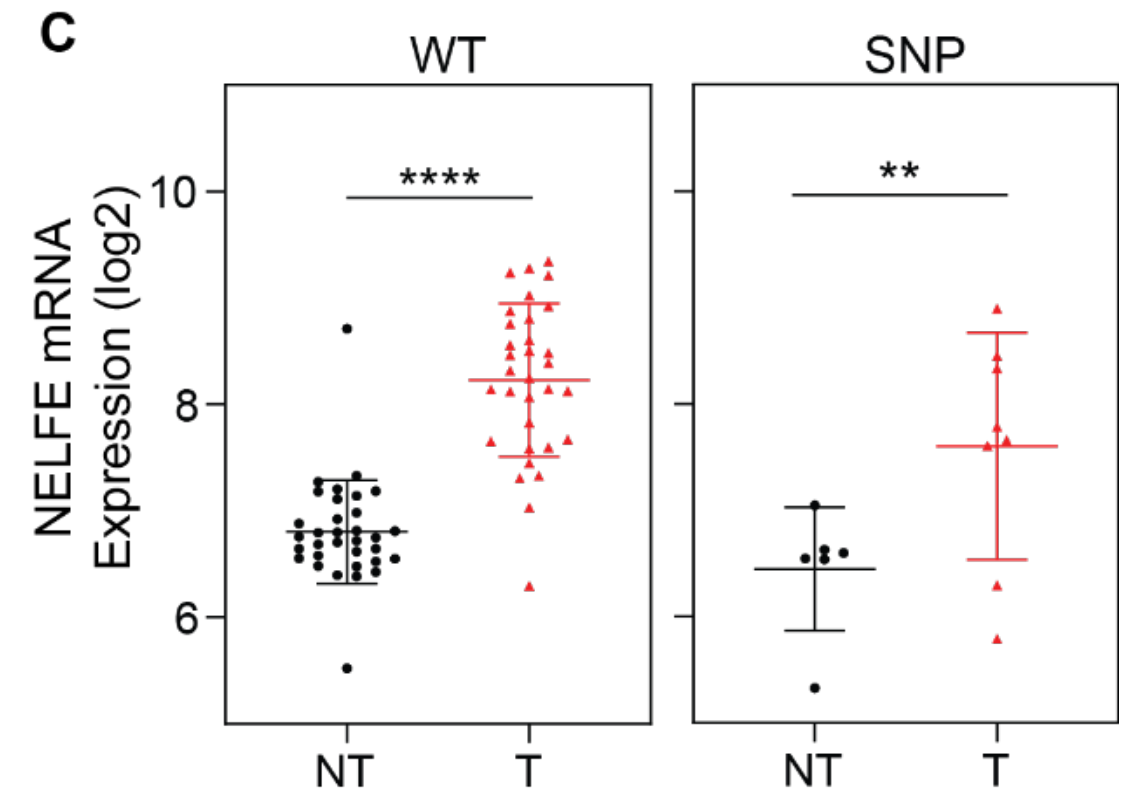

Figure S1. Rs79208225 SNP in HCC patients is associated with decreased *NELFE* mRNA expression. (A) Demographic and clinical variables of WT and rs79208225 SNP patient cohorts. **(B)** Kaplan-Meier 5-year overall survival curve for WT and rs79208225 SNP patients (WT n=73, SNP n=8). Statistical analysis was conducted by log-rank test. **(C)** TaqMan RT-qPCR to detect *NELFE* mRNA expression between tumor (T) and non-tumor (NT) tissue in WT (T n=32, NT n=33) and SNP (T n=8, NT n=6) patients. Data presented as mean±SD. Statistical analysis was conducted with a one-way ANOVA with Holm-Šídák's multiple comparisons test. **p< 0.01, ****p<0.0001

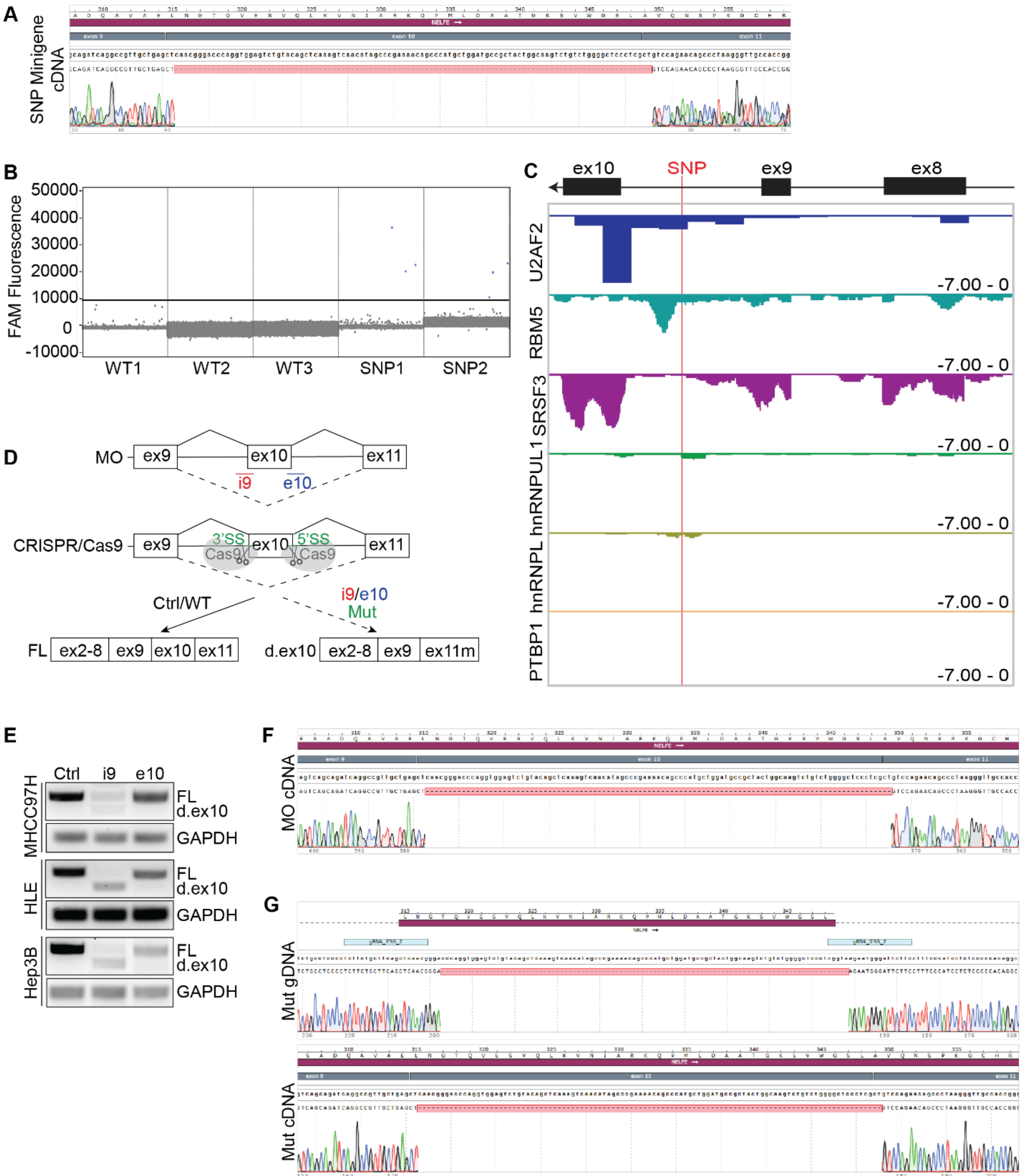

Figure S2. Rs79208225 SNP promotes alternative splicing of *NELFE* exon 10 producing an uncharacterized *NELFE* mRNA isoform. (A) Sanger sequencing of the bottom band from the SNP minigene RT-PCR in Fig. 2A showing exon 10 skipping. **(B)** Digital PCR data of d.ex10 isoform detection in WT and rs79208225 SNP patient tumor tissue. Each dot is a well. Threshold (black line) is 9500. **(C)** Crosslinking immunoprecipitation data from the ENCODE database of splicing related proteins visualized using integrative genomics viewer to identify proximity of their

binding regions to the rs79208225 locus (red line). **(D)** Schematics of the models used to study exon 10 loss: MO induced alternative splicing (i9 and e10) and CRISPR deleted exon 10 (Mut). Dotted line indicates exon 10 skipping. **(E)** RT-PCR agarose gels showing *NELFE* mRNA isoform switching and reduced full length (FL) 48 hours after treatment with MOs in HCC cell lines. **(F)** Alignment of the d.ex10 band from i9 MO RT-PCR showing exon 10 skipping. **(G)** Alignment of Huh1 d.ex10 mut genomic DNA (top) and cDNA (bottom) sequencing with reference *NELFE* gene and RNA sequences, respectively.

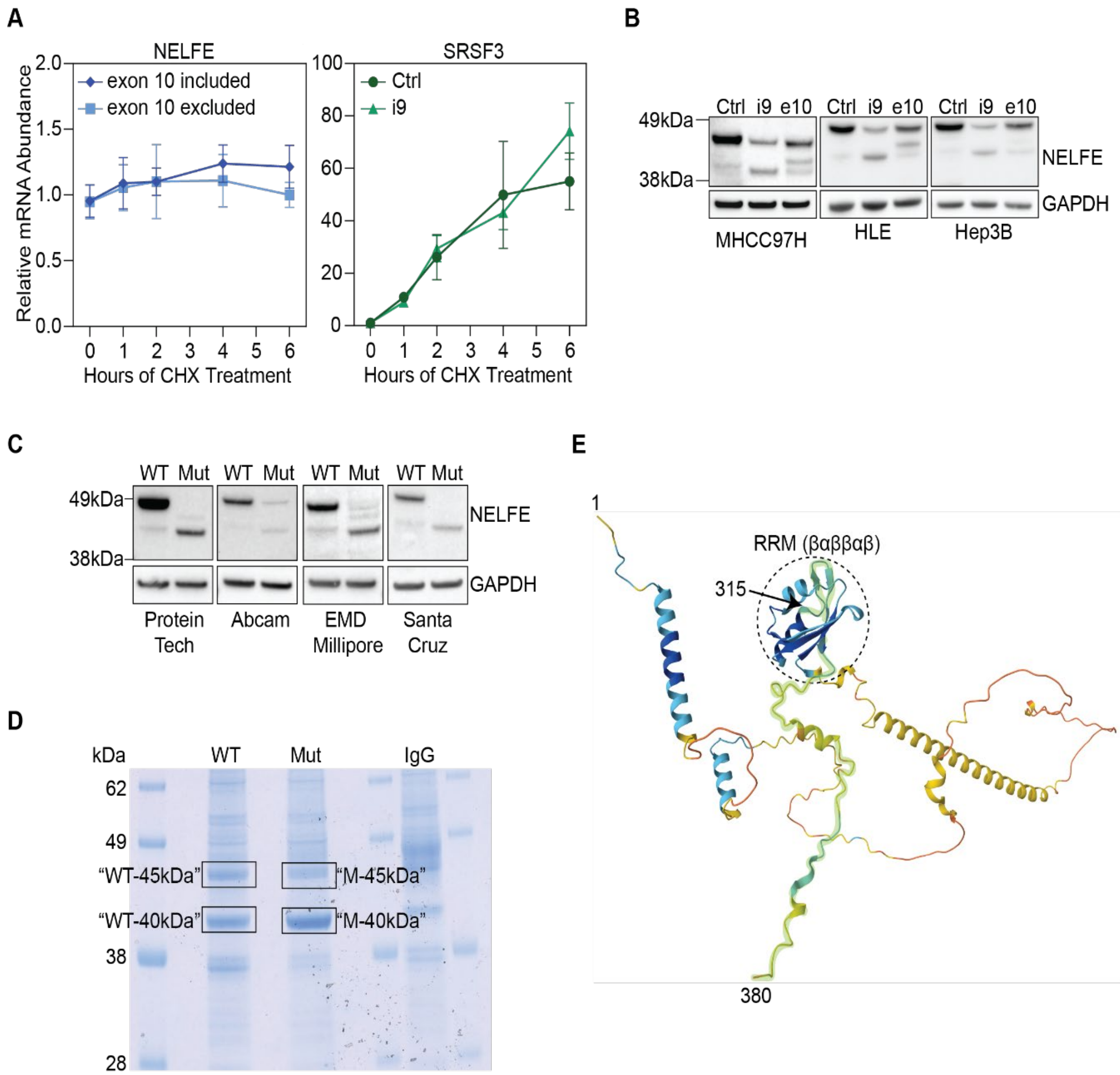

Figure S3. *NELFE* exon 10 skipping produces an uncharacterized protein isoform. (A) RT-qPCR analysis of mRNA abundance after treatment with 0.1mg/mL cycloheximide (CHX) to evaluate if d.ex10 undergoes a translation associated mRNA decay pathway. Left, relative abundance of exon 10 included mRNA from ctrl MO treated cells and exon 10 excluded mRNA from i9 treated cells. Cells were treated with CHX 48 hours after MO transfection. Exon 10 included transcripts were detected using a primer that spans the exon9-exon10 junction. Exon 10 excluded transcripts were detected using a primer that spans the exon9-exon11 junction. Primers detecting the poison exon in SRSF3 were utilized as a positive experimental control. **(B)** Representative immunoblots of NELFE after 72 hours of MO treatment in HCC cell lines. **(C)** Immunoblots of NELFE in Huh1 WT and Huh1 d.ex10 mut cells using multiple NELFE antibodies. **(D)** Representative scan of pre-cut gel submitted for mass spectrometry. Black boxes indicate bands analyzed. Three biological replicates were analyzed. **(E)** AlphaFold prediction for WT NELFE (380aa) folding. The RRM is in the dotted circle. The arrow points to aa315, where the d.ex10 isoform begins to differ and the highlighted region depicts the sequence that differs in d.ex10.

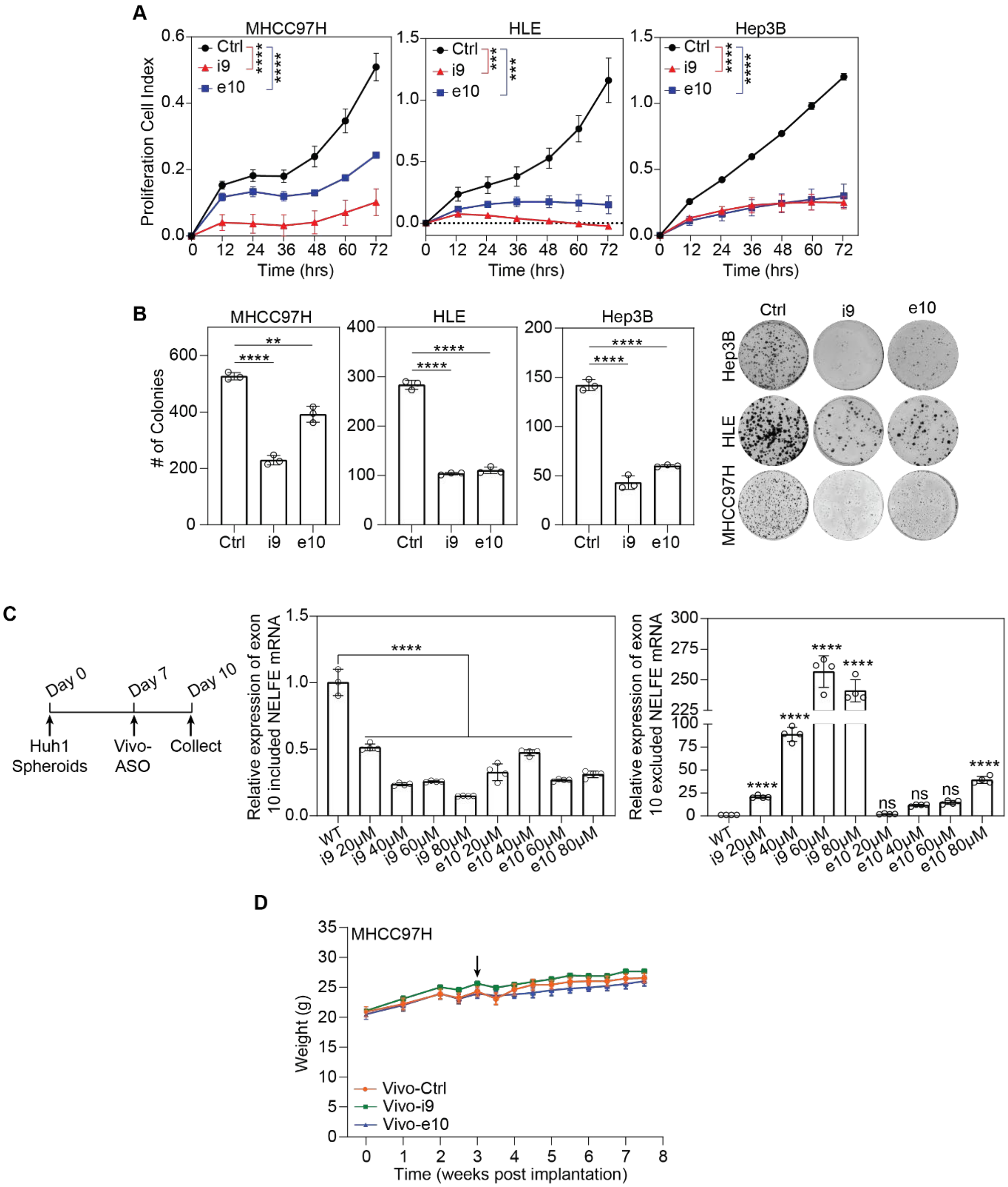

Figure S4. NELFE MOs reduce cancer cell phenotypes across HCC cell lines. (A) Cell proliferation assay in MO treated HCC cell lines. Cells were plated for xCELLigence 48 hours after MO transfection and measured every 12 hours for 72 hours. Data presented as mean± SD (n=3-4). Statistical analysis was conducted at 72 hours with a Student's t-test. ***p<0.001 and ****p<0.0001. **(B)** Cell colony formation assay in MO treated HCC cell lines. Cells were plated 48 hours after MO transfection. Left, quantification of colony formation after 10-14 days. Data

presented as mean ± SD (n=3). Statistical analysis was conducted with a Student's t-test. **p<0.01 and ****p<0.0001. Right, representative crystal violet stains. **(C)** Left, experimental timeline of identifying optimal vivo-MO dosage on spheroids. 150,000 Huh1 WT cells were incubated in an algimatrix for 1 week then treated with vivo-MO for 72 hours. Right, RT-qPCR of exon 10 included and excluded isoforms in the different vivo-MO concentration treated spheroids. Exon 10 included transcripts were detected with a TaqMan probe targeting the exon10-exon 11 junction. Exon 10 excluded transcripts were detected with a TaqMan probe targeting the exon9-exon11 junction. Ct values were normalized to the endogenous control 18S. Data presented as mean ± SD. Statistical analysis was conducted using a one-way ANOVA with Dunnett's multiple comparisons test. ns=not significant and ****p<0.0001. **(D)** Weights of MHCC97H implanted athymic nude mice over the duration of the experiment. Arrow indicates when treatment began. Data are presented as mean ± SEM (vivo-Ctrl n=5, vivo-i9 n=6, vivo-e10 n=5). Statistical analysis was conducted using a two-way ANOVA with Dunnett's multiple comparisons test.

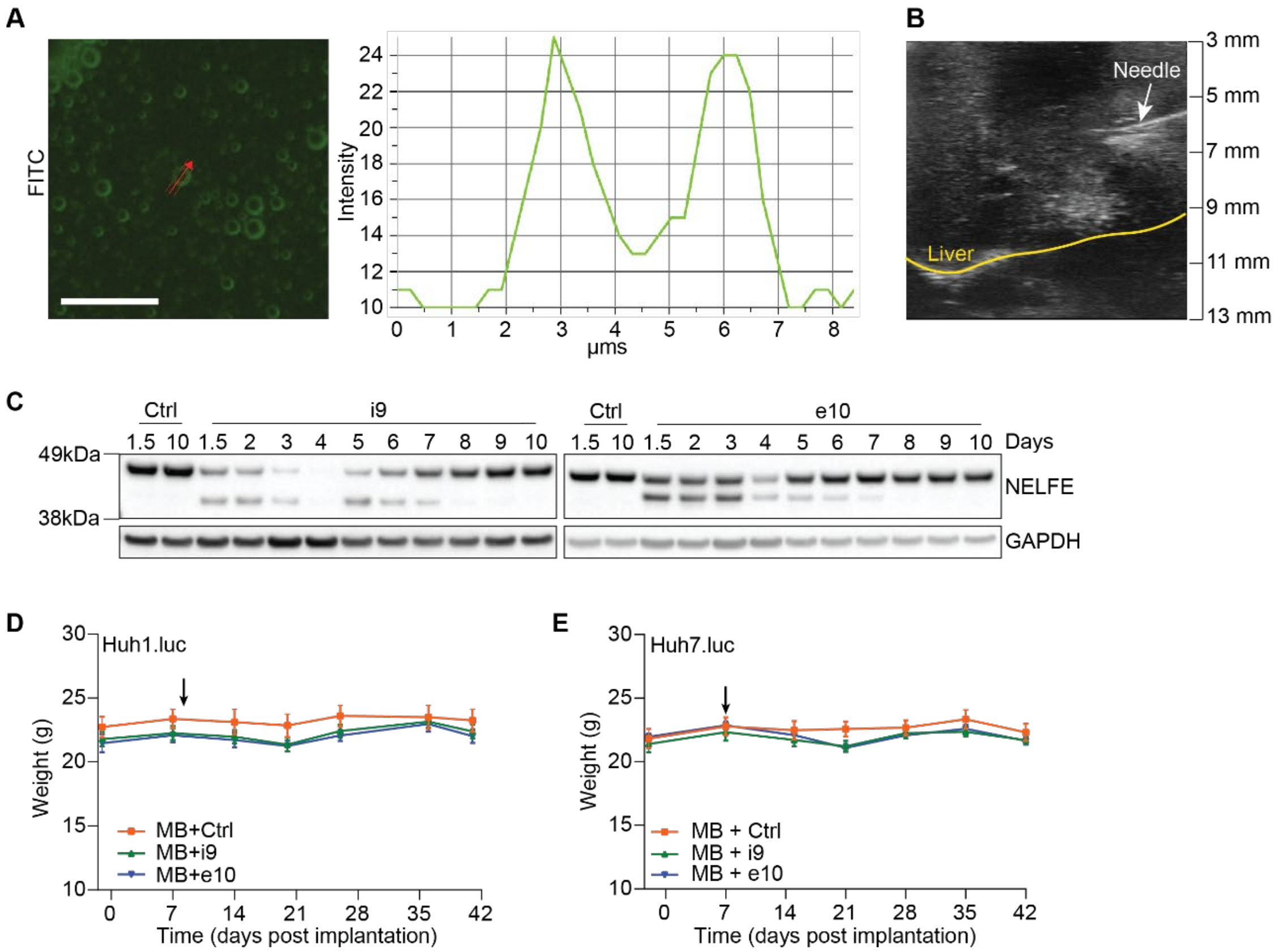

Figure S5. Ultrasound-targeted MBs loaded with NELFE MOs do not affect mice body weight. (A) Quantification of fluorescence across a single MB. Left, a representative FITC image with the red arrow indicating the analyzed section. Scale bar is 20μm. Right, quantification of intensity of fluorescence over analyzed region. The two peaks indicate in increased intensity as bubbles coat the outside. **(B)** Image of ultrasound guided HCC cell injections. **(C)** Immunoblots of NELFE expression over ten days of i9 MO or e10 MO treatment. **(D and E)** Weights of Huh1.luc **(D)** and Huh7.luc **(E)** mice over the treatment weeks of the vivo-MO+MB mice studies. Black arrow indicates when treatment began. Data are presented as mean ± SEM. Statistical analysis was conducted using a two-way ANOVA with Dunnett's multiple comparisons test.

Table S1. Proteomics Results. Mass spectrometry analysis of anti-NELFE immunoprecipitates from Huh1 WT and Huh1 d.ex10 mutant cells, with normal rabbit IgG as the negative control. The complete dataset is provided as a separate Excel file.

Table S2. Cell Line Characteristics.

Cell Line	Histological Type	Race	Age	Sex	HBV	HCV	rs79208225	*NELFE* Copy Number	p53	TERT
Huh1	HCC	Asian	53	M	Y	N	N	3	p.Arg110Leu	
HLE	HCC	Asian	68	M	N	N	N	3	p.Gly244Ala	c.1-124C>T
Hep3B2.1-7	HCC	African American	8	M	Y	N	N	3	null	
Huh7	HCC	Asian	57	M	N	Y	N	2	p.Tyr220Cys	
MHCC97H	HCC	Asian	39	M	Y	N	N	2	p.Glu51Ter	

Table S3. Primer sequences.

Name	Sequence	Experiments
SNP Seq F	GCCGTTGCTGAGGTTGGA	SNP Genotyping
SNP Seq R	TCGGGCTATGTTGACTTTGA	SNP Genotyping
NELFE SNP F2 Long	AGATCTGCCGCCGCGATCGCTGTGCCTTCGTCACCTATGAAAAG	Minigene RT-PCR
pLenti-c-seq R	GATGCCGTAGCAGAGGGTGGT	Minigene RT-PCR, RIP-qPCR
NELFE SNP F2	GGTCGGATTCATTCCCTGAACG	Isoform Switching RT-PCR, Total NELFE RT-qPCR
NELFE SNP R2	GCCATCCACAAGGTTTTCCTTG	Isoform Switching RT-PCR
Trunct1+2 Seq R	CCATGGAGAGGTCAATGATG	Total NELFE RT-qPCR
GAPDH F	TGCACCACCAACTGCTTAGC	Isoform Switching RT-PCR, Total NELFE RT-qPCR
GAPDH R	GGCATGGACTGTGGTCATGAG	Isoform Switching RT-PCR, Total NELFE RT-qPCR
ex8 F	ACATCATTGACCTCTCCATGGAC	CHX RT-qPCR
ex9ex10 R	TCCCGTTGAGCTCAGCAAC	CHX RT-qPCR
cDNAisoformR2	TGTTCTGGACAGCTCAGCAA	CHX RT-qPCR
SRSF3 F	CGTGTAAGAGTGGAACTGTCG	CHX RT-qPCR
SRSF3 poison R	ATGATGGTGACTCTGCGACGA	CHX RT-qPCR
